# CAXII Is a Surrogate Marker for Luminal Breast Tumors Regulated by ER and GATA3

**DOI:** 10.3390/cancers14215453

**Published:** 2022-11-06

**Authors:** Lucas Porras, Faustine Gorse, Ndeye Khady Thiombane, Louis Gaboury, Sylvie Mader

**Affiliations:** 1 Institute for Research in Immunology and Cancer, Faculty of Medicine, Université de Montréal, Montréal, QC H3C 3J7, Canada; 2 Faculty of Pharmaceutical Sciences, University of British Columbia, Vancouver, BC V6T 1Z3, Canada; 3 Department of Pathology and Cell Biology, Faculty of Medicine, Université de Montréal, Montréal, QC H3C 3J7, Canada

**Keywords:** breast cancer, luminal tumors, ERα, PR, CA12/CAXII, GATA3, estrogen signaling, ER status, immunostaining

## Abstract

**Simple Summary:**

Breast cancer is a heterogeneous disease and treatment needs to be adapted to individual tumors. Two thirds of breast tumors may benefit from treatment with drugs targeting a specific protein, the estrogen receptor alpha, a regulator of gene expression activated by female sex hormones. However, a significant percentage of tumors will recur and progress. To help detect more accurately the expression status and activity of this protein, a gene that it upregulates, the progesterone receptor (PR), is routinely used in the clinic as an additional marker. Here, we show that PR status is an imperfect reflection of the expression and/or overall activity of estrogen receptor alpha and identify another marker that can perform this task more consistently. Overall, use of CAXII as a marker of ER^+^ tumors should reinforce the diagnosis of ER status and prediction of its activity and enhance the accuracy of hormonal therapy delivery.

**Abstract:**

Estrogen receptor alpha (ERα) expression in ~2/3 breast tumors selects patients for hormonal therapies. Tumors negative for ERα but positive for the progesterone receptor (PR, encoded by *PGR*) have also been candidates for ER-targeting therapies, as PR expression may reflect undetected ER activity. Conversely, PR^−^ status in ER^+^ tumors predicts a worse therapeutic response. Our analysis of breast tumor transcriptome datasets, however, revealed that in tumors with lower *PGR* expression, the clinical PR status does not correlate accurately with the expression of *ESR1* or of ER target genes, including *PGR* itself. We identified carbonic anhydrase 12 (*CA12*) as an estrogen target gene better correlated with *ESR1* than *PGR*, reflecting *CA12* regulation by both ERα and the luminal factor and upstream *ESR1* regulator GATA3. Immunostaining supported strong positive correlations at the protein level with ERα and GATA3 in a cohort of 118 tumors. Most ER^+^PR^−^ tumors expressed CAXII at levels similar to those of ER^+^PR^+^ tumors, consistent with observations in tumor transcriptome datasets and with active estrogenic signaling in some ER^+^PR^−^ breast cancer cell lines. The few ER^−^PR^+^ tumors did not express CAXII or the other luminal markers FOXA1 and GATA3. Overall, CAXII is a luminal marker that can help interpret ER status in single ER/PR positive tumors.

## 1. Introduction

Breast cancer is a complex and heterogeneous disease, as is apparent from the differential expression of therapeutic targets ERα and HER2, and by the identification of intrinsic subtypes based on whole tumor transcriptomic profiles. Different gene signatures have been proposed to identify these subtypes, resulting in various classification schemes [1,2,3,4,5]. In spite of variations between classifiers, luminal subtypes essentially correspond to ERα-positive (ER^+^) tumors and display the highest prevalence, representing together more than 70% of all malignancies. ERα, a ligand-dependent transcription factor and member of the nuclear receptor family, is the main therapeutic target in these tumors as it drives cell proliferation and survival. Targeted treatments include anti-estrogens (AEs), which modulate ERα activity and/or accelerate its turnover, and aromatase inhibitors (AIs), which prevent the synthesis of estrogens [6]. Both ERα and PR, encoded by *PGR*, a target gene of ERα, are used as markers of tumors that may benefit from hormonal therapies [7,8,9,10], their expression at the protein level being routinely detected by immunohistochemistry (IHC). ERα and PR protein levels in ER^+^ tumors are often stronger and more homogeneous than in adjacent normal tissue, where they are heterogeneous within luminal cells of the mammary ducts and alveoli [11]. However, ER^+^ tumors can also display intra-tumor heterogeneity for ERα and PR expression levels. The threshold for positivity for both markers is nuclear expression in at least 1% of the cells according to the 2010 American Society of Clinical Oncology (ASCO) guidelines [12], but can be as high as 10% in clinical practice [13]. ER^−^ tumors include both tumors over-expressing the *ERBB2* gene, which codes for the ERBB2/HER2 membrane receptor, and those negative for HER2 as well as ERα and PR (triple negative, TN).

The causes of intra-tumoral heterogeneity of ER expression are currently still speculative and could correspond to the co-existence of different tumor cell states or of different biological subtypes, the latter potentially resulting in intrinsic resistance to hormonal treatment. Low ERα expression (1–10% cells) has indeed been associated with increased rates of relapse and progression on hormonal therapies [14,15,16,17], and may result in an unstable ER status during tumor progression [14]. The St Gallen international expert consensus on the primary therapy of early breast cancer 2015 describes ER values between 1 and 9% as equivocal [18]. A 2020 update from ASCO recommends use of a new reporting category for these tumors, ER Low Positive, with additional steps to confirm and interpret the results [19]. Although detection of PR as well as ER reinforces recommendation of hormonal therapies in double hormone receptor (HR) positive tumors (55–67% of all tumors), especially at the low end of the ER expression spectrum, it also yields single positive tumors, mainly ER^+^PR^−^ tumors (7–16% of all tumors) and a small fraction of ER^−^PR^+^ tumors (1–4%) [9,20]. The ER^−^PR^+^ tumor category has been proposed to reflect staining artifacts, warranting reanalysis of the ER^−^ diagnosis [21,22]. However, others have found that ER^−^PR^+^ tumors are a rare but distinct entity [23,24,25]. These tumors were reported to have survival rates and incidence of *BRCA* mutations similar to ER^−^PR^−^ tumors, with which they share phenotypic traits [10,26,27,28]. Nevertheless, ER^−^PR^+^ tumors have been traditionally considered for hormonal therapies and even included in the Luminal A group in the absence of *ERBB2* amplification in surrogate tumor subtype classifications [10]. On the other hand, ER^+^PR^−^ tumors have 5-yr survival rates intermediate between those of ER^+^PR^+^ and ER^−^PR^−^ tumors, conferring a prognostic value to PR expression [8,9,20]. The contribution of PR expression to prognosis has been attributed to its role as a surrogate marker of ERα expression/activity [13,29,30] and also to the direct contribution of PR isoforms to tumor cell biology and progression [31,32,33]. ER^+^PR^−^ tumors, nevertheless, are still candidates for hormonal therapies, in association or not with chemotherapy, as response rates are lower but not null in this tumor group [34].

Apart from *PGR,* other estrogen target genes have been considered for their association with ER status and/or prediction of response to hormonal therapies, including *pS2*/*TFF1* [35] and Growth Regulation by Estrogen in Breast cancer 1 (*GREB1*) [36]. Contrary to *PGR*, neither are included in the PAM50 classifier [3], although both are part of the 256 genes CIT breast cancer classifier [5]. They also play roles in ER^+^ tumorigenesis that may confound their significance as ER surrogate markers. The TFF1 protein was reported to stimulate breast cancer cell migration [37] and to exert apoptosis-protecting effects in doxorubicin-treated cells [38]. Gene deletion in mice reduced breast tumorigenesis [39]. Nevertheless, high intratumoral expression of TFF1 was found to have a low but significant predictive value for response to tamoxifen [40]. GREB1 has been reported to act as an ERα coactivator, but also to modify it by glycosylation, to increase its stability and decrease response to tamoxifen [41,42]. It may also have ER-independent roles as a proliferation regulator in breast cancer [43]. In ovarian cancer, it acts as a tumor promoter, promoting cell proliferation, migration and mesenchymal morphology [44]. It was also identified as a target of the Wnt/β-catenin pathway required for hepatoblastoma progression [45].

Another estrogen target gene known to be enriched in ER^+^ tumors is *CA12*, encoding the membrane protein carbonic anhydrase 12 (CAXII) [46,47], one of the 15 members of the carbonic anhydrase family catalyzing the hydration of carbon dioxide [48]. This family of metalloenzymes includes a total of four classes depending on their subcellular location [48]. CAXII belongs to the class of proteins found at the plasma membrane [49]. Regulation of *CA12* by estrogens has been observed in MCF-7 and T-47D breast cancer cells, and ChIP experiments have indicated binding of ERα to an enhancer at −6 kb [46]. Although *CA12* is regulated in breast cancer cells by other transcription factors (TFs) such as AP2γ and by hypoxia, albeit much less than its paralog *CA9* [50,51], we show that its RNA expression levels are amongst the most highly correlated with those of the *ESR1* gene in several transcriptome datasets. We confirmed that ERα is a major regulator of *CA12* expression, acting via two ERE-containing enhancers, and that the luminal factor GATA3, itself an upstream regulator of the *ESR1* gene [52], also induces *CA12* expression in ER^+^ tumors. We further validated the strong association of CAXII and ERα positivity at the protein level in a 118-tumor cohort. Comparison with PR status indicated that CAXII was broadly expressed in ER^+^PR^−^ tumors but was absent from ER^−^PR^+^ tumors. We propose that CAXII may usefully complement PR for the determination of ERα expression and activity.

## 2. Materials and Methods

### 2.1. Cell Culture Conditions

Breast cell lines were purchased from the American Type Culture Collection (ATCC) and maintained in a humidified 37 °C, 5% CO_2_ incubator. MCF-7, SKBR-3 and MDA-MB-453 breast cancer cells were cultured in Dulbecco’s Minimal Eagle’s Medium (DMEM; Wisent Inc., Saint-Jean Baptiste, QC, Canada) supplemented with 10% fetal bovine serum (FBS; Sigma, St. Louis, MO, USA) and 1% penicillin/streptomycin (P/S, Wisent Inc., Saint-Jean Baptiste, QC, Canada). MDA-MB-231 were maintained in DMEM with 5% FBS and 1% P/S. T-47D, ZR-75-1 and HCC-70 cells were maintained in RPMI 1640 (Wisent Inc., Saint-Jean Baptiste, QC, Canada) with 10% FBS, 1% P/S, 10 mM HEPES pH 7.0 (BioBasic, Markham, ON, Canada), and 1% sodium pyruvate (Wisent Inc., Saint-Jean Baptiste, QC, Canada). MCF-10A cells were maintained in Dulbecco’s Modified Eagle’s Medium F-12 (DMEM F-12; Wisent Inc., Saint-Jean Baptiste, QC, Canada) without calcium chloride with 10% FBS and 1% P/S. For estrogenic treatments, cells were cultured in hormone-depleted media (without phenol red and supplemented with charcoal-dextran treated FBS (FBST)).

### 2.2. RNA Extraction, Reverse Transcription and Real-Time Quantitative PCR

Total RNA was prepared using 1 mL of QIAzol Lysis Reagent (QIAGEN, Hilden, Germany), 0.2 mL of chloroform (Sigma-Aldrich, St. Louis, MO, USA) and 0.5 mL isopropanol 99.5% (Fisher, Hampton, NH, USA) per cell pellet. RNA aliquots (1 µg) were reverse-transcribed using the RevertAid H first minus strand cDNA synthesis kit (Thermo Fisher Scientific, Waltham, MA, USA) with oligo(dT)_18_ primers. Expression levels of target genes were assessed by real-time quantitative PCR (qPCR) using the Universal ProbeLibrary system (Roche, Basel, Switzerland) and the ViiA 7 Real-Time PCR System (ThermoFisher Scientific, Waltham, MA, USA) using *YWHAZ* and *RPLP0* as housekeeping genes. RT-qPCR primer sequences and UPL probe numbers are listed in Supplementary Table S1.

### 2.3. Protein Extraction and Western Blotting

Whole cell lysates were prepared using RIPA lysis buffer (Tris-HCl pH 7.5 50 mM, NaCl 150 mM, Triton X-100 1%, SDS 0.1%, sodium deoxycholate 0.5% and freshly added protease inhibitors (PMSF 10 mM and leupeptin, pepstatin, and aprotinin, 1 µg/mL)). Extracts were homogenized for 10 min before sonication (4 °C, 10 min; Bioruptor, Diagenode, Denville, NJ, USA) and quantified using a Lowry assay (BioRad, Hercules, CA, USA). Equal amounts of proteins (20–40 µg) were lysed in Laemmli blue 1X and electrophoresed on an 8% SDS-polyacrylamide gel at 80V for 2 h. Proteins were transferred on a PVDF membrane using a Trans-Blot Turbo Transfer System (BioRad, Hercules, CA, USA). Membranes were blocked using PBS-T (PBS 1X, Tween 20 0.05%) with 5% milk for 1h and blotted with rabbit or mouse antibodies directed against CAXII, ERα, FOXA1, GATA3, β-actin or lamin B1 overnight (see Supplementary Table S2), and then incubated with horseradish peroxidase–conjugated secondary anti-mouse or anti-rabbit IgG (Cedarlane, Burlington, Canada) for 45 min. Immunodetection was performed using the Clarity Western ECL substrate (BioRad, Hercules, CA, USA) for enhanced chemiluminescence. Original blots see supplementary File S1.

### 2.4. siRNA Transfection

Cells were maintained in hormone-depleted media for three days before transfection. Cells were seeded and transfected when attached. A SMARTpool of four siRNAs was used against *CA12* and *TFAP2C* and two different ON-TARGETplus siRNAs against *ESR1, FOXA1* or *GATA3* (Dharmacon, Lafayette, CO, USA). One ON-TARGETplus Non-Targeting siRNA (“si-Control”, Dharmacon, Lafayette, CO, USA) was used as a negative control. Transfection was performed with each siRNA (40 nM) using the SilentFect reagent (BioRad, Hercules, CA, USA) for a total duration of 72 h. Cells were then treated or not with E2 (25 nM) or with vehicle (EtOH 0.024%), 24 h before cell collection for subsequent RNA and protein extractions. Sequences of all siRNAs are provided in Supplementary Table S3.

### 2.5. Chromatin Immunoprecipitation (ChIP)

Cells maintained in a hormone-depleted medium for three days were either treated or not with E2 (25 nM) for 1 h before crosslinking at room temperature by addition of formaldehyde (1%) for 10 min. Crosslinking was stopped by adding glycine (0.125 M) for 5 min and washing cells twice with ice-cold PBS 1X. Collected cells were lysed on ice using lysis buffer (Tris-HCl pH 8.0 10 mM, EDTA 10 mM, EGTA 0.5 mM, Triton X-100 0.25%, and protease inhibitors) for 5 min. After centrifugation, cell pellets were washed with a second lysis buffer (Tris pH 8.0 10 mM, NaCl 200 mM, EDTA 1 mM, EGTA 0.5 mM and protease inhibitors), incubated for 30 min and centrifugated. Cells resuspended in sonication buffer (Tris pH 8.0 10 mM, NaCl 140 mM, EDTA 1 mM, EGTA 0.5 mM, SDS 0.5%, Triton X-100 0.5%, sodium deoxycholate 0.05%, and protease inhibitors) were sonicated using a Bioruptor (Diagenode, Denville, NJ, USA; maximum intensity, 30 s intervals between pulses). IP was performed on sonicated chromatin prepared from three million cells by addition of 3 µg of each antibody (see Supplementary Table S2) with a 1:1 mix of Dynabeads A and G (Invitrogen, Waltham, MA, USA) in ChIP dilution buffer (Tris pH 8.0 10 mM, NaCl 150 mM, EDTA 2 mM, Triton X-100 1%). Mixes were then incubated on a rotor O/N at 4 °C to capture antibody-protein-DNA complexes. Beads were washed with buffer (Tris pH 8.0 20 mM, EDTA 2 mM, Triton X-100 1% and SDS 0.1%) containing decreasing concentrations of NaCl (from 500 to 50 mM). Input DNA and IP samples were decrosslinked (NaHCO3 10 mM, SDS 1%) O/N at 65 °C. Eluates were subsequently incubated with RNase A (BioBasic, Markham, ON, Canada) for 30 min at 65 °C and Proteinase K (ThermoScientific, Waltham, MA, USA) for 1 h at 65 °C. Finally, DNA fragments were purified on EZ-10 columns (BioBasic, Markham, ON, Canada). ChIP results are shown as percentage of input relative to IgG (% input with antibody/% input with IgG). ChIP qPCR primer sequences and the respective TaqMan probe numbers are provided in Supplementary Table S4.

### 2.6. Tissue Microarrays and Immunohistochemitry

Tissue micro-arrays (TMAs) were prepared from cores (1 mm) extracted from each of 118 formalin-fixed and paraffin-embedded (FFPE) breast tumor tissues as previously described [53]. These samples were obtained from patients with primary breast tumors who underwent surgery at the Hôtel Dieu Hospital and at the Centre Hospitalier de l’Université de Montréal (CHUM) between 2001 and 2018. The study was conducted according to the guidelines of the Declaration of Helsinki and approved by the CHUM ethics committee (SL 05-019). Tumor characteristics extracted from the pathological reports include ER, PR and HER2 status as well as histological classification and grades (Supplementary Table S5).

Sections (4 µm) were prepared from TMAs or FFPE normal mammary gland tissues. Deparaffinization and antigen retrieval were performed using a Discovery XT automatic stainer (Ventana Medical Systems, Oro Valley, AZ, USA) and incubated with primary antibodies directed against CAXII, ERα, FOXA1 or GATA3 (see Supplementary Table S6 for antibodies and dilutions). Single staining was performed using Bond polymer DAB refine kits (#DS9800, Leica Biosystems, Wetzlar, Germany) on a Bond RX stainer (Leica Biosystems, Buffalo Grove, IL, USA). Dual staining was performed by staining CAXII as above, and then using the Green Chromogen (DC9913, Leica Biosystems, Wetzlar, Germany) in conjunction with BOND Polymer Refine HRP PLEX Detection (#DS9914) for ERα, FOXA1 or GATA3. The sections were then stained with Gill hematoxylin to visualize nuclei. Stained tissue sections were scanned using the C9600 NanoZoomer System (Hamamatsu Corporation, Bridgewater, NJ, USA). The NDP Scan software (version 2.2.9; Hamamatsu Corporation, Bridgewater, NJ, USA) was used to extract all images. The stained tissue sections were scored using the QuPath software (version 0.3.0, https://github.com/qupath/qupath/releases, accessed on 26 September 2022). Training was performed on several cores for cell identification based on hematoxylin intensity staining and nuclei shapes, and for scoring of each marker based on a range of expression levels (from null to high expression) in tumor cells. QuPath provides the proportion of positive cells (0 to 5 scale) and staining intensities (0 to 3 scale), with a maximum total score of 8 [54]. HRP staining was analyzed in the nuclear or membrane compartment for ERα, FOXA1 and GATA3 or CAXII, respectively. Score cut-offs were set based on the trough in the bi-modal histogram representation for each protein (scores ≥ 4 for ERα, ≥ 3 for CAXII). IHC and co-IHC staining conditions for CAXII, ERα, GATA3 and FOXA1 and controls are listed in Supplementary Tables S6 and S7.

## 3. Results

### 3.1. The Clinical PR Status Is Not an Accurate Predictor of ESR1 Expression Levels or of ERα Activity

**Figure 1:**
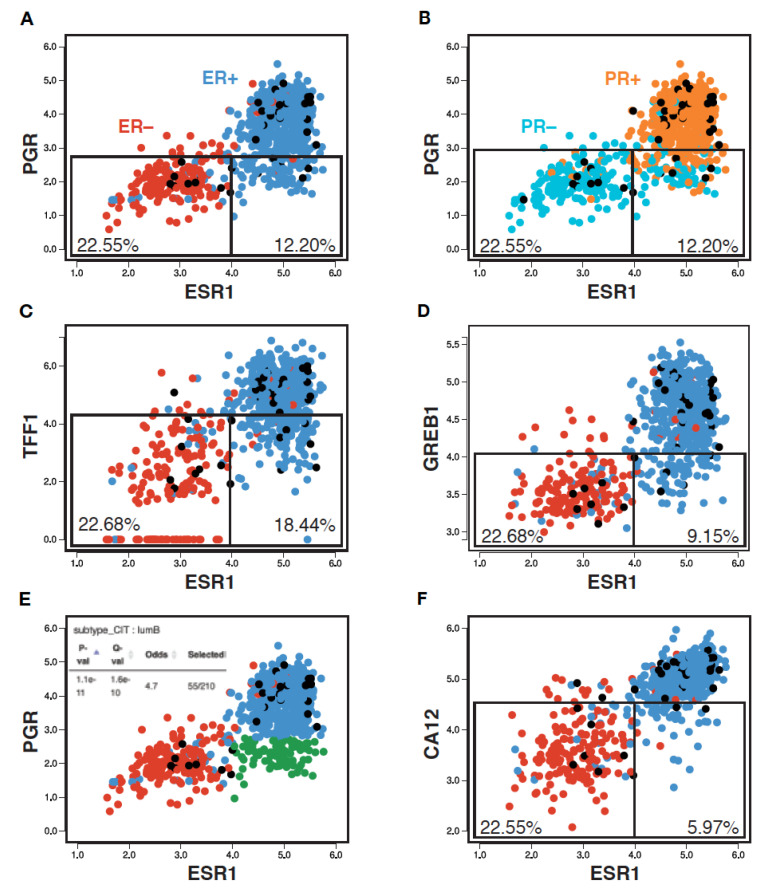
PR status imperfectly reflects *ESR1* expression, ER status and activity. Pair-wise scatterplots for the expression of *ESR1* vs *PGR* (**A**,**B**,**E**), *TFF1* (**C**), *GREB1* (**D**) and *CA12* (**F**) generated using MiSTIC [55]. Boxes identify 90% of *ESR1*^low^ tumors, and the portion of *ESR1*^high^ tumors identified using the same threshold of positivity for different ER target genes. Percentages of total tumors are shown. Enrichment of *ESR1*^high^*PGR*^low^ tumors (highlighted in green) in the CIT luminal B tumor subtype is shown in the inset of panel (**E**).

To examine whether the PR clinical status reflects ER expression and/or activity, we generated a pair-wise scatterplot representation of *ESR1* and *PGR* expression levels in an RNA-seq breast tumor transcriptome dataset from the Cancer Genome Atlas (TCGA, 754 tumors) using MiSTIC, a visualization platform for gene–gene correlation studies and enrichment analysis [55]. This analysis revealed that, although *PGR* expression levels were well correlated with those of *ESR1* (Pearson correlation 0.68), tumors with low *PGR* expression levels have a broad range of *ESR1* expression levels. Indeed, selecting a *PGR* expression level cut-off leading to the exclusion of ~90% of *ESR1*^low^ tumors (~22.5% of all tumors; Figure 1A, left box) excluded many tumors with high *ESR1* expression levels (12.2% of all tumors, ~16.3% of *ESR1*^high^ tumors; Figure 1A, right box). Note that the *ESR1* expression levels correlated well with ER status, with only 5.4% of tumors having a discrepant status (Figure 1A, ER^+^ tumors in blue, ER^−^ tumors in red). On the other hand, *PGR* expression levels displayed more discrepancies with the PR clinical status (13.4% overall; Figure 1B, PR^+^ tumors in orange, PR^−^ tumors in cyan). This may suggest either post-transcriptional regulation of *PGR* expression, or variability in calling PR status, especially for tumors with low to intermediate RNA levels (Figure 1B, boxes).

Other well-characterized ER target genes such as *TFF1* or *GREB1* (Figure 1C,D) displayed similar correlation coefficients with *ESR1* at the RNA expression level (0.70 and 0.68, resp., Figure S1). The overlap in expression levels between *ESR1*^high^ and *ESR1*^low^ tumors was higher for *TFF1* (exclusion of 18.4% of total tumors, i.e., 24.6% of *ESR1*^high^ tumors for an expression cut-off excluding 90% of *ESR1*^low^ tumors), but lower for *GREB1* (exclusion of 9.2% total tumors, i.e., 12.3% of *ESR1*^high^ tumors). Notably, a significant fraction of the *ESR1*^high^*PGR*^low^ tumors (green dots in Figure S2A) express high levels of *TFF1* (~75.3%) and/or *GREB1* (~77.4) (purple dots in Figure S2B,C). Conversely, most of the rare *ESR1*^low^*PGR*^high^ tumors (18 tumors, ~2.4%) express low levels of these other ER target genes (14/18 *TFF1*^low^, 15/18 *GREB1*^low^, not shown).

Together, these results indicate that PR scores do not appear to provide highly accurate information on either *ESR1* expression levels or ERα activity. Rather, *PGR* expression may reflect the activity of other regulatory pathways with prognostic value, as the *ESR1*^high^*PGR*^low^ tumor group was enriched in LumB tumors (Figure 1E, Q-value 1.6 × 10^−10^; Figure 1).

### 3.2. CA12 mRNA Levels Correlate with Those of Luminal Transcription Factor Genes ESR1, GATA3 and FOXA1 in Breast Tumor Transcriptome Datasets

**Figure 2:**
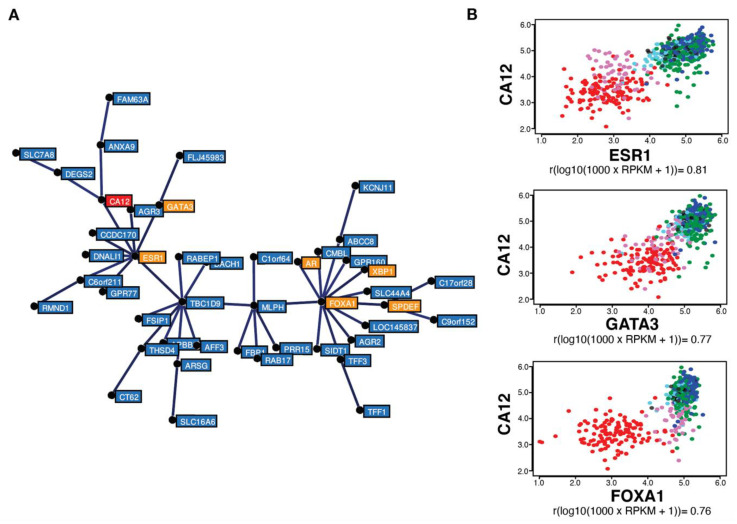
*CA12* mRNA levels are highly correlated with those of *ESR1*, *FOXA1* and *GATA3* in breast tumors. (**A**) Minimum spanning tree representing a cluster of genes expressed preferentially in luminal and molecular apocrine breast tumors, identified by gene correlation analysis from a TCGA breast cancer transcriptomic dataset comprising 754 tumors using MiSTIC software [55]. Genes encoding transcription factors (*ESR1*, *FOXA1*, *GATA3, SPDEF*, *XBP1* and *AR*) are highlighted in orange, whereas *CA12* is indicated in red. (**B**) Pair-wise correlation scatterplots for *CA12* and *ESR1* (top), *GATA3* (middle) or *FOXA1* (bottom) expression levels in the same cohort, with CIT tumor subtypes highlighted by different colors. Luminal A tumors appear in dark blue, luminal B in green, luminal C in light blue, molecular apocrine in magenta and basal-like in red. Normal-like or non-classified tumors are shown in black. Pearson correlation coefficients are indicated at the bottom of each pair-wise correlation scatterplot.

Gene correlation analysis in the same TCGA breast tumor dataset (754 tumors) using MiSTIC revealed a cluster of genes with highly correlated expression biased for luminal tumors (Figure 2A). The strong correlation of cluster genes is not the result of a copy number variation (CNV) event, as most genes are located on different chromosomes. This cluster includes six luminal transcription factor genes: *ESR1*, *FOXA1*, *GATA3*, *AR*, *SPDEF* and *XBP1* (in orange, Figure 2A), suggesting that clustering reflects transcriptional regulation. Apart from genes located proximal to and likely co-regulated with *ESR1* (*CCDC170*, *C6orf211*/ARMT1 and *RMND1*), known estrogen targets are also found in this cluster, including *TFF1*, *AGR3* and *CA12* (in red, Figure 2A). *CA12* encodes a carbonic anhydrase whose expression is regulated by estrogens in MCF-7 and T-47D breast cancer cell lines [46]. Interestingly, *ESR1* expression levels were more highly correlated with those of *CA12* (Pearson correlation coefficient of 0.81) than of *PGR*, *TFF1* and *GREB1*. *CA12* expression was also correlated, albeit to a slightly lower level, with luminal transcription factors *GATA3* and *FOXA1* (correlation coefficients of 0.77 and 0.76, respectively) (Figure 2B).

High correlation between *CA12* and *ESR1* expression is reproducible in the TCGA Firehose Legacy dataset comprising 960 breast tumor transcriptomes (Pearson correlation coefficient of 0.80, Figure S3A) and in the 1904 breast cancer samples from the METABRIC dataset (Pearson correlation coefficient of 0.79, Figure S3B). Outlier tumors in this correlation were those expressing low levels of *CA12* mRNA in spite of high *ESR1* levels, which were identified as a small fraction of luminal B tumors, and tumors expressing higher *CA12* mRNA levels than predicted from *ESR1* expression, representing a fraction of molecular apocrine (mApo) tumors in the CIT classification [5] (Figure 2B). mApo tumors are ER^–^ and express high levels of the androgen receptor (*AR*) and *FOXA1*. They include most ER^–^HER2^+^ and some TN tumors [56,57,58,59,60]. Interestingly, mApo tumors with high *CA12* levels also had high levels of *GATA3*, whereas other mApo tumors have *GATA3* levels comparable to those of basal-like tumors (Figure 2B). Basal-like tumors expressed low levels, whereas luminal tumors expressed high levels of both *ESR1* and *CA12* (Figure 2A,B). Thus, setting a threshold of *CA12* RNA positivity excluding 90% of *ESR1*^low^ tumors led to exclusion of a much lower number of *ESR1*^high^ tumors compared to *PGR* (about half, Figure 1F).

### 3.3. CAXII Is Detected Predominantly in ER^+^ Breast Cancer Cells

**Figure 3:**
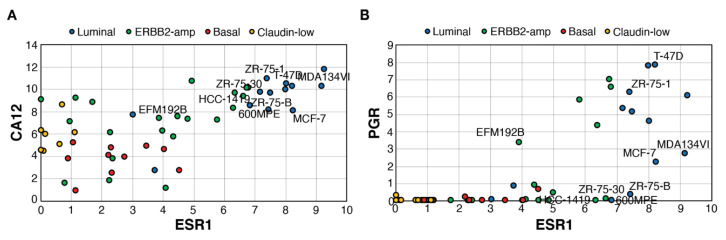
*ESR1*^high^ cell lines express high levels of *CA12*, but variable levels of *PGR*. Pair-wise scatterplots for the expression of *ESR1* vs those of *CA12* (**A**) or *PGR* (**B**). Values are Log2(Rpkm+1). RNA levels and cell line subtyping are from [61]. Selected cell lines are labeled.

Using a transcriptome dataset for 51 breast cancer cell lines including luminal, *ERBB2*-amplified, basal and claudin-low lines [61], we determined whether expression of *CA12* is also associated with that of *ESR1* (Figure 3A) and how it compares with *PGR* expression (Figure 3B). *CA12* expression at the RNA level was consistently high in all luminal cell lines. Basal tumors and claudin-low cell lines expressed lower levels of *CA12*. *ERBB2*-amp lines displayed a range of *ESR1* expression and *CA12* levels, with some overlap in *CA12* expression between *ESR1*^low^ and *ESR1*^high^ lines. *PGR* RNA levels were much lower in comparison, being absent in most claudin-low, basal and *ESR1*^low^ *ERBB2*-amp cell lines but also in some *ESR1*^high^ lines. Notably, as observed in tumors, several *ESR1*^high^*PGR*^null^ lines expressed high *CA12* levels. These lines, classified as luminal or *ERBB2*-amplified lines, include estrogen-responsive ZR75-30 and 600MPE lines [62], confirming that PR^−^ status does not necessarily reflect lack of estrogen response. Levels of *PGR* were also relatively low in estrogen-responsive MCF7 and MDA-MB-134VI [63].

Next, we compared levels of *CA12* RNA (RT-qPCR, Figure S4A) and CAXII protein (western analysis, Figure S4B) in cell lines corresponding to different breast cancer subtypes using a polyclonal antibody previously validated for CAXII detection in western and immunohistochemistry analyses [51,64]. MCF-7, T-47D and ZR-75-1 are luminal breast cancer lines, SKBR-3 and MDA-MB-453 are ER^−^ but express *FOXA1* and *AR* and are representative of the mApo subtype, with *ERBB2*-amplification and high expression in SKBR-3 and MDA-MB-453, respectively. HCC70 and MDA-MB-231 belong respectively to the basal-like and claudin-low subtypes of triple-negative breast cancer cell lines [5,65]. In addition, the MCF-10A cell line is immortalized but non-tumorigenic. *ESR1* RNA and ERα protein levels were, as expected, high in MCF-7, T-47D and ZR-75-1 cells and low in all other cell lines (Figure S4A,B). Strong RNA and protein expression of the *CA12* gene was detected in T-47D and ZR-75-1 cells and in the mApo SKBR-3 cell line. Weaker expression was observed in MCF-7 and in immortalized MCF-10A. Expression was low to undetectable in mApo MDA-MB-453, basal HCC70 cells and claudin-low MDA-MB-231 (Figure S4A,B). Western analysis further revealed the existence of multiple bands detected by the CAXII antibody. Different *CA12* transcripts are responsible for the translation of two main CAXII isoforms, including a longer form at 39 kDa (Gen-Bank accession # NM_001218.5) and a 37 kDa truncated isoform, resulting from translation of an RNA transcript lacking exon 9 (Gen-Bank accession # NM_206925.3). However, multiple glycosylation events result in different CAXII forms, including, for the longer isoform, a fully glycosylated form at 43 kDa [66]. Alternative splicing and variants presenting amino acid substitution at the level of glycosylation sites, such as p.His121Gln and p.Glu143Lys [66] could be responsible for the different ratios of CAXII isoforms. Migration patterns indicate that only ER^+^ breast cancer cells express the high-molecular weight CAXII form (upper band, Figure S4B).

Globally, the CAXII protein expression patterns mirrored those at the RNA level, confirming the specificity of the antibody and suggesting that post-transcriptional regulation does not play a major role in the control of CAXII protein expression in breast cancer cell lines.

### 3.4. CA12 Is Regulated by Luminal Transcription Factors ERα and GATA3 in ER^+^ Cell Lines

**Figure 4:**
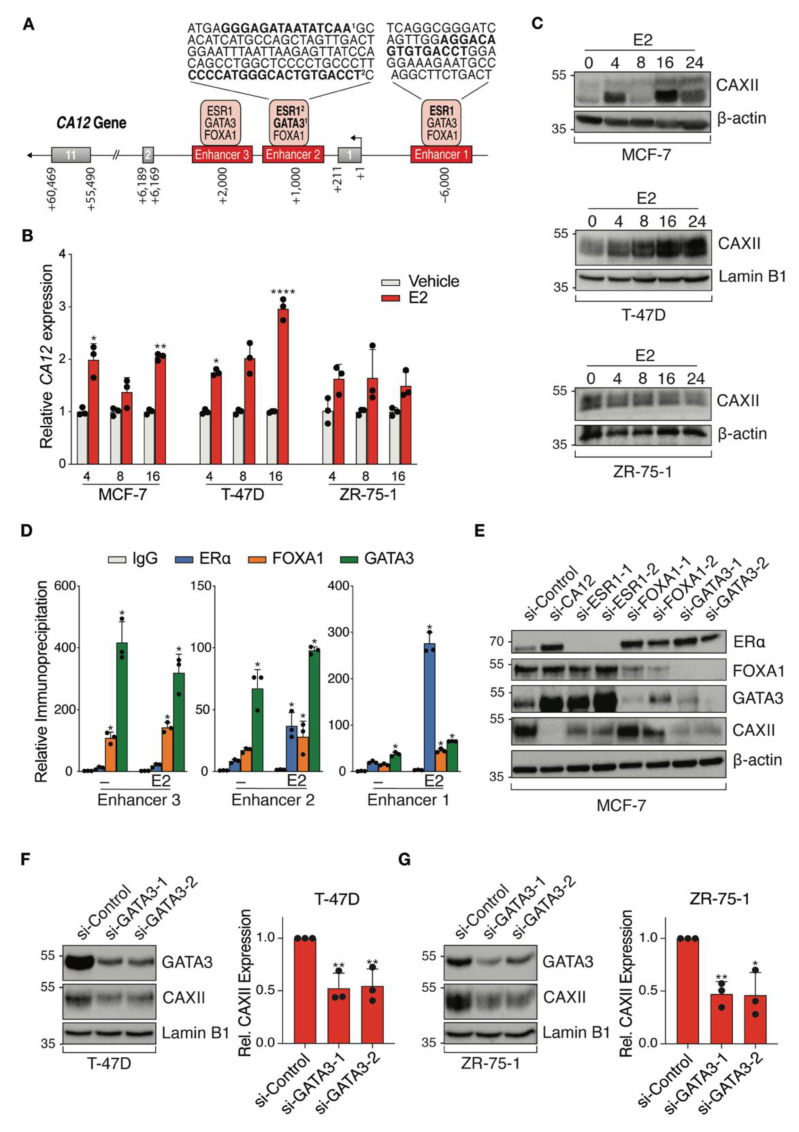
ERα and GATA3 positively regulate *CA12* expression in luminal breast cancer cell lines. (**A**) Schematic *CA12* gene organization with exons (grey boxes) and putative enhancers (red boxes). Predicted binding motifs for ERα and GATA3 are highlighted in bold in sequences for enhancers 1 and 2. (**B**) MCF-7, T-47D and ZR-75-1 cell lines were cultured in hormone-depleted medium for five days and then treated with E2 (25 nM). *CA12* mRNA levels were measured by RT-qPCR at 4, 8 and 16 h. *RPLP0* and *YWHAZ* were used as housekeeping genes for normalization. Values are presented as ratios over the expression in vehicle-treated samples for each time point. Asterisks indicate statistically significant regulations (Average of *n* = 3; *, *p* ≤ 0.05; **, *p* ≤ 0.01; ****, *p* ≤ 0.0001; Student’s unpaired *t*-test). (**C**) CAXII protein levels from MCF-7, T-47D and ZR-75-1 cells were analyzed at 0, 4, 8, 16 and 24 h after treatment with E2 by Western blotting (*n* = 3, a representative blot is shown). β-actin or Lamin B1 housekeeping proteins were used as loading controls. (**D**) MCF-7 cells were cultured in hormone-depleted medium for three days and then treated with E2 (25 nM) for 1 h before fixation and collection. Binding of ERα, FOXA1 and GATA3 to *CA12* enhancers was examined by ChIP-qPCR. Relative immunoprecipitation levels (ratios to IgG in vehicle-treated cells) are shown. The assay was performed twice with similar results. Asterisks indicate statistically significant binding compared to IgG in vehicle-treated cells for each enhancer from one experiment performed in triplicates (*, *p* ≤ 0.05; one-way ANOVA test, Dunnett’s multiple comparisons test). (**E**) MCF-7 cells were cultured in hormone-depleted medium for three days and transfected with a SMARTpool of siRNAs targeting *CA12* and two different siRNAs targeting *ESR1*, *FOXA1* or *GATA3*. Cells were then collected two days after transfection. Protein levels of CAXII, ERα, FOXA1 and GATA3 were analyzed by Western blotting (*n* = 2). β-actin was used as a loading control. (**F**,**G**) T-47D and ZR-75-1 cells were cultured in hormone-depleted medium for three days and transfected with two different siRNAs targeting *GATA3*. Cells were then collected two days after transfection. Protein levels of CAXII and GATA3 were analyzed by Western blotting in T-47D (**F**) and ZR-75-1 (**G**) cells; CAXII levels were quantified for both cell lines using ImageJ (average of *n* = 3 for quantification, one representative blot shown; *, *p* ≤ 0.05; **, *p* ≤ 0.01; Student’s unpaired *t*-test). Lamin B1 was used as a loading control.

ENCODE ChIP-Seq datasets visualized on the UCSC Genome Browser identified *CA12* regulatory regions bound by luminal transcription factors with associated binding motifs (Figure 4A). TFs ERα, FOXA1 and GATA3 bound three potential *CA12* enhancers, with predicted estrogen response elements (ERE) motifs in enhancers 1 and 2 and predicted GATA3 binding motifs in enhancer 2. Regulation of *CA12* by estrogens in MCF-7 and T-47D cells was previously described and binding to enhancer 1 was confirmed by ChIP-qPCR [46]. Accordingly, we observed time-dependent induction of *CA12* upon treatment of MCF-7 and T-47D cells with 25 nM 17β-estradiol (E2), with maximal expression at 16 h, but did not observe induction of *CA12* by E2 in ZR-75-1 cells within 24 h (Figure 4B). These results were reproduced at the protein level, CAXII expression in these cells being maximal at 16 h in MCF-7 and at 24 h in T-47D cells but remaining mostly stable in ZR-75-1 cells (Figure 4C). Further, ChIP-qPCR confirmed estradiol-induced recruitment of ERα on enhancers 1 and 2, compared to control IgG in MCF-7 cells (Figure 4D) and to gene desert regions (not shown).

We also confirmed GATA3 recruitment as well as that of FOXA1 on all three enhancers in MCF-7 cells (Figure 4D). To further explore regulation of *CA12* by luminal transcription factors, we targeted *ESR1*, *FOXA1* and *GATA3* by siRNAs (2 siRNAs per gene) as well as *CA12* (siRNA pool) in MCF-7 cells cultured in a hormone-depleted medium (Figure 4E). These experiments revealed that GATA3 in addition to ERα regulates CAXII protein expression. As GATA3 acts in a positive regulatory loop with ERα [52], GATA3 could be involved in the regulation of *CA12* indirectly. However, ERα levels were not significantly depleted by siRNAs against GATA3 (or conversely) in MCF-7 cells under our experimental conditions. To support the evidence of regulation of *CA12* by GATA3, we transfected siRNAs against GATA3 in two other cell lines, T-47D (Figure 4F) and ZR-75-1 (Figure 4G). The results showed a significant decreased expression of CAXII, supported by a quantification of the protein levels using Image J.

Taken together, these results indicate that *CA12* is a luminal gene that is regulated by ERα and GATA3, although potential regulation by FOXA1 in some settings cannot be excluded.

### 3.5. CAXII Expression Is Increased with Lack of Polarity in Invasive ER^+^ Tumors

**Figure 5:**
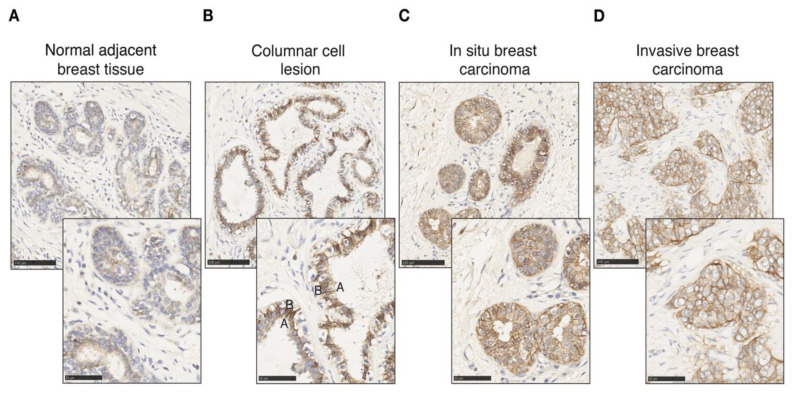
CAXII expression is increased in tumoral epithelial cells with loss of polarity. CAXII staining (in brown) of normal adjacent mammary tissue (**A**), columnar cell lesion (**B**), in situ breast ductal carcinoma (**C**) and invasive breast ductal carcinoma (**D**) at 20× and 40× magnification. The apical (A) and basal (B) poles of a luminal cell are labeled.

*CA12* is expressed physiologically in epithelial cells in the pancreas, kidney and colon as well as in the mammary gland [48,67,68]. We probed CAXII levels in breast tumors as well as in normal tissue by immunohistochemistry (IHC). Different controls were used to validate and optimize CAXII staining of tissue sections, including the pancreas, kidney and colon as positive expression controls and lymph node, liver and spleen for negative controls (Supplementary Figure S5). CAXII levels are higher in breast ductal carcinoma in situ compared to normal lobules and ducts [51]. In pulmonary adenocarcinomas, increased CAXII levels correlate with tumor grade and aggressiveness [69]. Accordingly, in normal mammary gland and normal tumor-adjacent mammary tissues, CAXII levels, detected by IHC using the same antibody as above, were low and heterogeneous (Figure 5A), similar to those of ERα. Conversely, in breast tumors, CAXII levels were high and homogeneous. The osmotic exchange of bicarbonates by CAXII occurs at the basolateral level and not in the lumen in renal and pancreatic epithelial cells [67,68]. CAXII basolateral polarity was observable in columnar cell lesions, in which bilayer integrity is preserved (Figure 5B) but was lost in intra-ductal proliferating cells in ductal carcinoma in situ (DCIS) (Figure 5C) and in cells migrating into the stromal compartment in invasive ductal carcinoma (IDC) (Figure 5D).

### 3.6. Levels of CAXII Correlate with Those of ERα, GATA3 and FOXA1 in Breast Tumor Arrays

**Figure 6:**
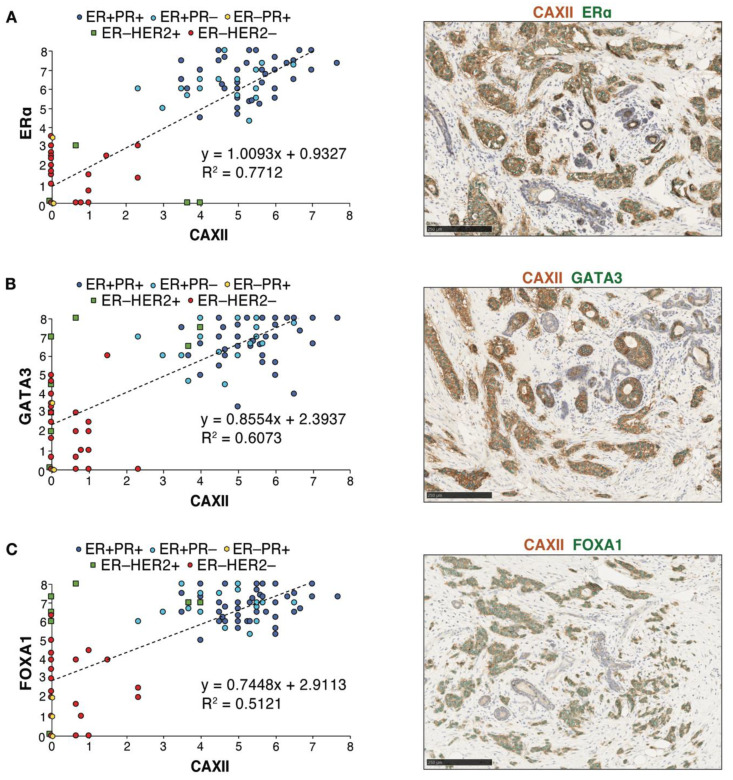
Protein expression levels of CAXII and of the luminal transcription factors ERα, FOXA1 and GATA3 are correlated in breast tumors. Correlations between CAXII and ERα (**A**), GATA3 (**B**) or FOXA1 (**C**) expression scores are shown for all tumors in the TMAs. Scores were computed by QuPath using membrane (CAXII) and nuclear signal intensities (ERα, GATA3, FOXA1). ER^+^PR^+^ tumors are highlighted in dark blue circles, ER^+^PR^−^ tumors in light blue circles, ER^−^PR^+^ tumors in yellow diamonds, ER^−^HER2^+^ tumors in green squares and ER^−^HER2^−^ tumors in red circles. Representative co-staining for CAXII (brown) and each of these luminal transcription factors (green) is also shown.

Immunohistochemistry analysis of ERα, GATA3, FOXA1 and CAXII was then carried out in consecutive slices of seven tissue micro-arrays (TMAs) displaying 118 tumors represented in triplicates. Scores for nuclear (for ERα, GATA3 and FOXA1) or membrane (for CAXII) staining in individual tumors were computed using QuPath [54] after training for identification of epithelial, stromal and immune compartments and validation by a pathologist (Figure S6A,B). Cut-offs for positivity were set at 4 for ERα and at 3 for CAXII, based on their bimodal distributions (Figure S7A,B). QuPath scores for ERα were largely concordant with the ER clinical status. Linear regression analysis of score distributions for CAXII and ERα, GATA3 or FOXA1 revealed a high determination coefficient (R^2^ ~0.77, ~0.61 and ~0.51, resp., Figure 6A–C), supporting at the protein level the correlations observed at the RNA level between *CA12* and *ESR1*, *GATA3* or *FOXA1* expression. Accordingly, co-staining of CAXII (membrane localization, brown) and ERα, GATA3 or FOXA1 (nuclear localization, green) in tumors further supported their co-expression (Figure 6A–C).

### 3.7. CAXII Is Mainly Expressed in ER^+^ Breast Tumors

**Figure 7:**
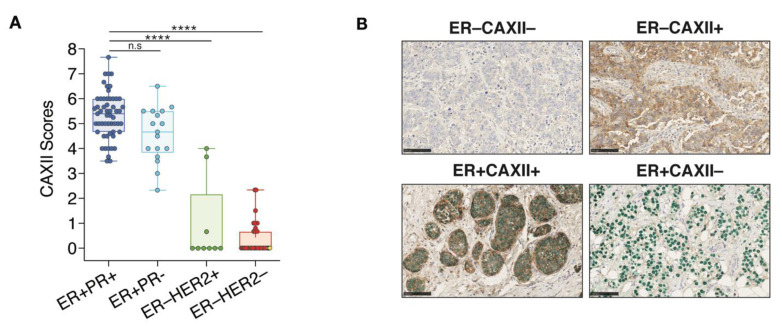
CAXII expression discriminates ER^+^ from ER^−^ breast tumors. (**A**) Boxplot representing CAXII score distribution according to ER, PR and HER2 status (7 arrays, *n* = 118; ns, *p* > 0.05; ****, *p* ≤ 0.0001; Dunn’s multiple comparisons post-hoc test performed after Kruskall-Wallis test (*p* < 0.0001)). ER^−^PR^+^ tumors are highlighted in yellow in the ER^−^HER2^−^ group. (**B**) Representative cores for the four observed phenotypes of CAXII and ER status after co-staining with CAXII (brown) and ERα (green). Scores were computed by QuPath using membrane (CAXII) and nuclear (ERα) signal intensities.

CAXII score distributions within each clinical tumor subtype indicate that in 72 ER^+^ tumors, 71 (98.6%) were positive for CAXII. In these tumors, the 55 ER^+^PR^+^ and the 17 ER^+^PR^−^ have significantly higher scores for CAXII expression compared to ER^−^HER2^+^ and TN clinical tumors (Figure 7A). However, no significant difference in CAXII scores was observed between ER^+^PR^+^ and ER^+^PR^−^ tumors. The only ER^+^ tumor negative for CAXII (score 2.3) is also negative for PR, suggesting low or absent ERα activity. Conversely, 7 out 9 ER^−^HER2^+^ tumors and all 34 TN tumors (ER^−^PR^−^HER2^−^) are negative for CAXII expression (Figure 7A). The 2 ER^−^HER2^+^ tumors positive for CAXII express intermediate CAXII levels (scores at 3.67 and 4.0). These tumors also express FOXA1 and GATA3 [70], but are negative for PR. The 3 tumors classified as ER^−^PR^+^ in the clinic (ER scores below 1%, PR scores between 1–10%) were negative for both ERα and CAXII in our analysis (yellow diamonds in Figure 6A, yellow dots in the ER^−^HER2^−^ group in Figure 7A). These tumors were also all negative for expression of GATA3 and FOXA1 (Figure 6B,C). Two out of 3 tumors expressed high levels of the basal marker FOXC1 (not shown), suggesting a dominant basal-like phenotype. The third one was null for all subtype markers assessed, suggestive of a different TN subtype. Representative staining results for ER and CAXII expression are shown from co-staining corresponding to the two most frequently observed phenotypes, ER^+^CAXII^+^ (71 tumors) and ER^−^CAXII^−^ (37 tumors), and the two rarer phenotypes ER^−^CAXII^+^ (2 tumors, both HER2^+^) and ER^+^CAXII^−^ (1 tumor) (Figure 7B).

## 4. Discussion

ER status remains the main diagnostic factor for selection of tumors for hormonal therapies. Lack of PR expression in ER^+^ tumors is a bad prognosis factor and may be used as an indication for additional chemotherapy, whereas its expression in the absence of ER may prompt re-examination of ER staining and inclusion of controls without clear guidelines as to whether or not hormonal therapies may be of benefit. Additional markers that could reinforce the diagnosis of ER expression and activity are thus desirable. In this study, we identified *CA12* as one of the ERα target genes whose RNA expression is most correlated with that of *ESR1*, *GATA3* and *FOXA1* in breast cancer datasets. This high degree of correlation at the RNA level stems both from the very high correlation of *ESR1* RNA and protein levels and the regulation of *CA12* by both ERα and by GATA3, which may cooperate by synergizing for recruitment of coactivators or of the basal transcription machinery, and via cross-regulation [52,71,72,73,74]. Our TMA study further confirmed the value of the CAXII protein as a marker of luminal tumors and of ERα activity in IHC assays. In particular, CAXII IHC status correlated better with ER clinical status than PR clinical status.

Our dataset contained three ER^−^ tumors with a positive but low PR score (1–10%), which would have led to the diagnosis of two tumors as PR^−^ if a 10% threshold was applied. CAXII was absent in all three ER^−^PR^+^ tumors, compatible with lack of ER expression. Low FOXA1 levels in all three tumors and high FOXC1 levels in two of them further suggest TN phenotypes. Thus, staining with CAXII in addition to ER and PR, and possibly in combination with subtype markers such as FOXA1 and FOXC1, may spare some patients with ER^−^PR^+^ tumors futile long-term hormonal therapy treatments, and emphasize the need for TNBC-targeting therapies and genetic testing for BRCA mutations.

A large fraction of ER^+^ tumors (23.6%, 17 out of 72) in our dataset had a negative PR status, in spite of the low threshold for PR positivity, and most of these (15/17) expressed ER in more than 10% of the cells. Strikingly, 16/17 ER^+^PR^−^ tumors were positive for CAXII, suggesting a luminal phenotype (supported by positive scores for FOXA1 and GATA3), and, potentially, active ER signaling. Observations at the RNA level in the TCGA dataset also support active ER signaling in an important fraction of ER^+^PR^−^ tumors. Indeed, the majority (~75%) of *ESR1*^high^*PGR*^low^ tumors (12.2% of all tumors) express high levels of another ER target gene (*GREB1*, *TFF1* or *CA12*), suggesting that few tumors express fully inactive ER proteins. Lack of PR positivity, but not of other ERα target genes, may result from differential activity of ERα on its different target genes, possibly due to variable levels of ER cofactors. Other explanations for the lack of PR expression in a subset of ER^+^ tumors include copy number loss or CpG island methylation, independent from ER regulation. Irrespective of the mechanism involved, loss of PR expression likely contributes to bad prognosis for reasons other than loss of ER signaling, including its own action as a transcriptional regulator and/or as a modulator of ER action [33]. In this respect, it would be important to determine whether the higher degree of discrepancy between *PGR* RNA expression and PR clinical status compared to *ESR1*/ERα results from scoring variability, affecting the accuracy of PR status as a prognostic biomarker, or from potential post-transcriptional regulation of PR levels, the significant entity for progesterone signaling.

In the TCGA dataset, RNA expression levels of *CA12* comparable to those in some ER^+^ tumors were observed in a subset of molecular apocrine tumors, which are ER^−^, but positive for AR and FOXA1 expression, and often *ERBB2*-amplified [56,57,58,59,60]. Two ER^−^HER2^+^FOXA1^+^ tumors indeed had moderate expression levels of CAXII in our study cohort. Although *CA12* expression is highly correlated with ER status in breast tumors at the RNA and protein levels, genes are rarely regulated by a single transcription factor. Other TFs such as GATA3 or AP2γ [70], or environmental factors such as hypoxia [49,50] may contribute to CAXII positivity in the absence of ER expression. This may result in one or several *CA12* enhancers remaining active in some tumors. Indeed, DNA methylation patterns on *CA12* enhancers 1 (at –6 kb) and 2 (+2 kb) were intermediate in two mApo cell lines (SKBR-3 and MDA-MB-453), with partial CpG methylation on one of the two regions, vs complete methylation in basal-like and claudin-low cell lines (HCC-1954, Hs578T and MDA-MB-231), and lack of methylation in luminal cell lines (MCF-7 and T-47D) [70]. Tumors expressing *CA12* in spite of lack of ERα are, however, likely to be negative for other markers of ER signaling such as PR, as was observed here for our two ER^−^CAXII^+^ tumors.

An advantage of CAXII as a biomarker is its membrane localization, enabling combining it usefully with nuclear ER detection in co-staining. In addition, the intra-tumoral heterogeneity of the ERα protein observed in several mammary tumors suggests that its expression is differentially regulated from one cell to another. Whether intra-tumor heterogeneity of ERα protein levels reflects the co-existence of different subtypes within the same tumor remains unclear. A surrogate marker of *ESR1* expression expressed on cell plasma membranes such as CAXII may enable the isolation of ER^+^ cells in heterogeneous tissues by fluorescence-activated cell sorting (FACS), and ultimately characterization of sorted tumor subpopulations via functional genomics approaches to determine genetic/epigenetic differences between these populations. CAXII may also be used in combination with other markers such as EpCAM and/or CD49f [75] to enhance purification of normal luminal mammary epithelial cells.

Limitations of this study include the small number of lobular carcinoma (nine samples) and absence of less frequent luminal subtypes such as tubular or mucinous tumors. It will be interesting in the future to explore whether the high correlation between *CA12* and *ESR1* remains valid in these subtypes. In addition, only three ER^−^PR^+^ cases were present in our cohort. This specific subgroup should be further studied in the future in larger cohorts, especially in retrospective studies in order to assess the usefulness of CAXII in distinguishing tumors that may benefit from hormonal therapies from those that do not. Similarly, further studies are needed to determine the frequency of ER^+^ tumors that are negative for both PR and CAXII (one tumor in our cohort, positive for FOXA1 and GATA3), and whether these tumors are also negative for other ER target genes and are unresponsive to hormonal therapies.

## 5. Conclusions

Altogether, CAXII is a luminal marker that would likely prove useful in conjunction with ERα and PR to identify tumors that may benefit from hormonal therapies, with or without adjuvant chemotherapy. CAXII may in addition prove useful to identify and sort heterogeneous cell populations in tumors and normal tissues.

## Data Availability

Public datasets analyzed for figure generation are referenced in the figure legends (cBioportal: https://www.cbioportal.org; MiSTIC: http://mistic.iric.ca. Data generated during this study is available upon request.

## Supplementary Materials

The following supporting information can be downloaded at: https://www.mdpi.com/article/10.3390/cancers14215453/s1, Figure S1: Correlations between mRNA levels of *ESR1* and of several estrogen target genes in breast cancer. Figure S2: *ESR1*^high^*PGR*^low^ status does not imply lack of ER signaling. Figure S3: *CA12* is mainly expressed in luminal tumors. Figure S4. *CA12* is highly expressed in luminal and mApo breast cancer cell lines. Figure S5. CAXII staining optimization. Figure S6. Tumor cell detection and scoring by QuPath in breast tumor sections. Figure S7. Bimodal distribution of CAXII and ER QuPath scores. Table S1. RT-qPCR primer sequences. Table S2. Immunoblotting and immunoprecipitation conditions. Table S3. SiRNA oligonucleotide sequences. Table S4. ChIP-qPCR primer sequences. Table S5. Clinicopathological characteristics of the 118 breast tumors. Table S6. Antibodies and IHC staining conditions. Table S7. Co-IHC staining conditions. File S1: Original blots.
