# Supplementary material for: CAXII Is a Surrogate Marker for Luminal Breast Tumors Regulated by ER and GATA3
Source: Cancers (Basel). 2022 Nov 6;14(21):5453. doi: 10.3390/cancers14215453 (PMC9657334; doi:10.3390/cancers14215453)

Blots figures and compiled data analysis

DMEM (MCF-7), RPMI (T-47D et ZR-75) complemented with 10% of FBST + 1%P/S

Gel SDS-PAGE : 8-10%

Loading quantity : 40µg

Standardisation : Lamin B1/Actin B

Blocking with 5% milk in PBST (Tris Buffer Saline 1X + 0.1% tween 20) for 1h.

Secondary Antibody dilution, anti-rabbit/mouse (1:10000)

Primary Antibodies

rAb CA12 (44 kDa), Dr Sly, dilution 1/3000 MCF-7

rAb CA12 (44 kDa), Dr Sly, dilution 1/7000 T-47D/ZR-75

Actin B (42 kDa), dilution 1/10000.

Lamin B1 (66kDa), dilution 1/3000.

Figure 4C

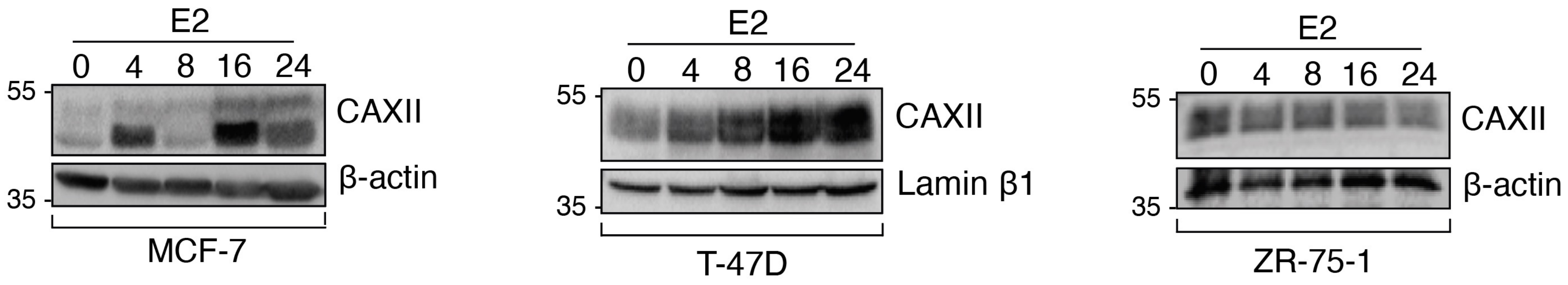

DMEM (MCF-7), RPMI (T-47D et ZR-75) complemented with 10% of FBST + 1%P/S

Gel SDS-PAGE : 8-10%

Loading quantity : 40µg

Standardisation : Lamin B1/Actin B

Blocking with 5% milk in PBST (Tris Buffer Saline 1X + 0.1% tween 20) for 1h.

Secondary Antibody dilution, anti-rabbit/mouse (1:10000)

Primary Antibodies

rAb CA12 (44 kDa), Dr Sly, dilution 1/3000 MCF-7

rAb CA12 (44 kDa), Dr Sly, dilution 1/7000 T-47D/ZR-75

Actin B (42 kDa), dilution 1/10000.

Lamin B1 (66kDa), dilution 1/3000.

Figure 4C

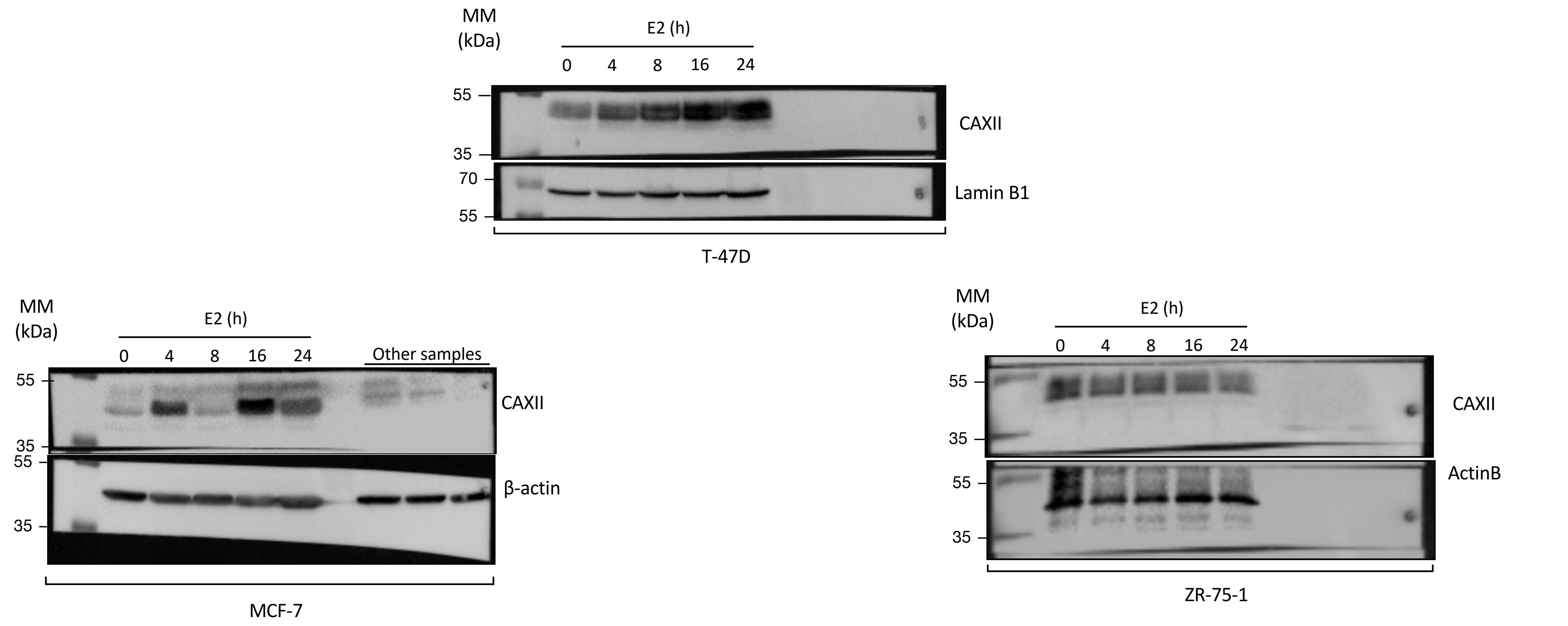

Figure 4D

First Replicate :

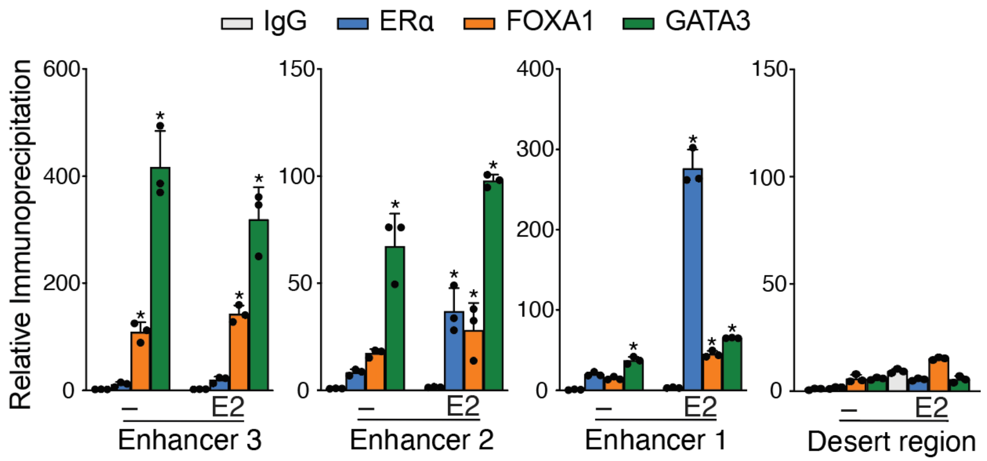

Second Replicate :

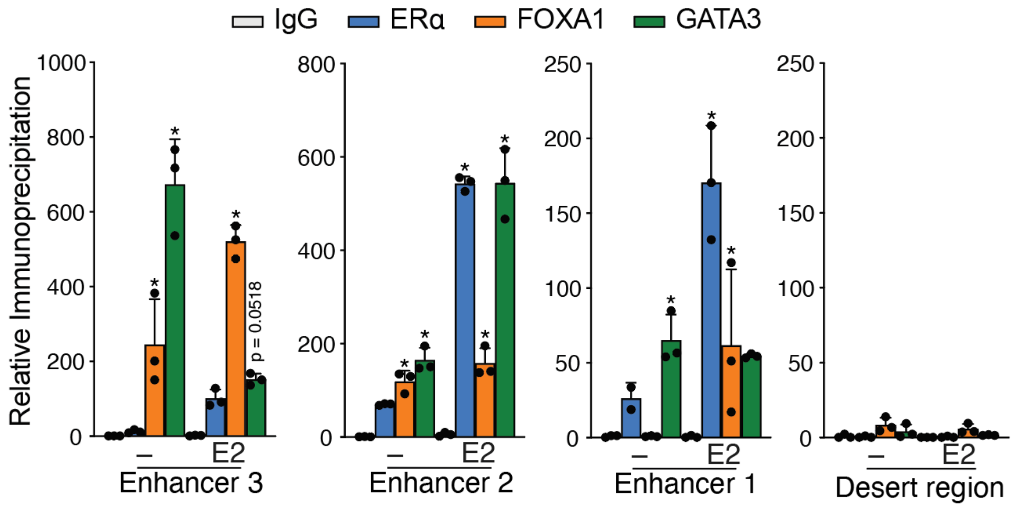

DMEM (MCF-7) complemented with 10% of FBST + 1%P/S

Gel SDS-PAGE : 8-10%

Loading quantity : 40µg
Standardisation : Actin B

Blocking with 5% milk in PBST (Tris Buffer Saline 1X + 0.1% tween 20) for 1h.

Primary Antibodies
rAb CA12 (44 kDa), Dr Sly, dilution 1/7000
rAb ERa (66 kDa), 60-C anticorps, dilution 1/2000
rAb FOXA1 (55kDa), Ab170933, dilution 1/1000
mAb GATA3 (48kDa), 558686 BD Pharmingen, dilution 1/1000
mAb Actin B (42 kDa), dilution 1/10000.

Secondary Antibody dilution, anti-rabbit/mouse (1:10000)

Figure 4E

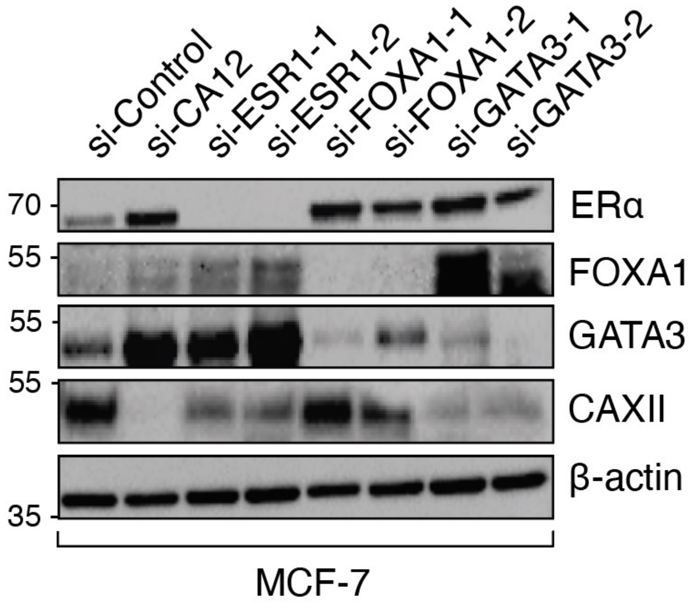

DMEM (MCF-7) complemented with 10% of FBST + 1%P/S

Figure 4E

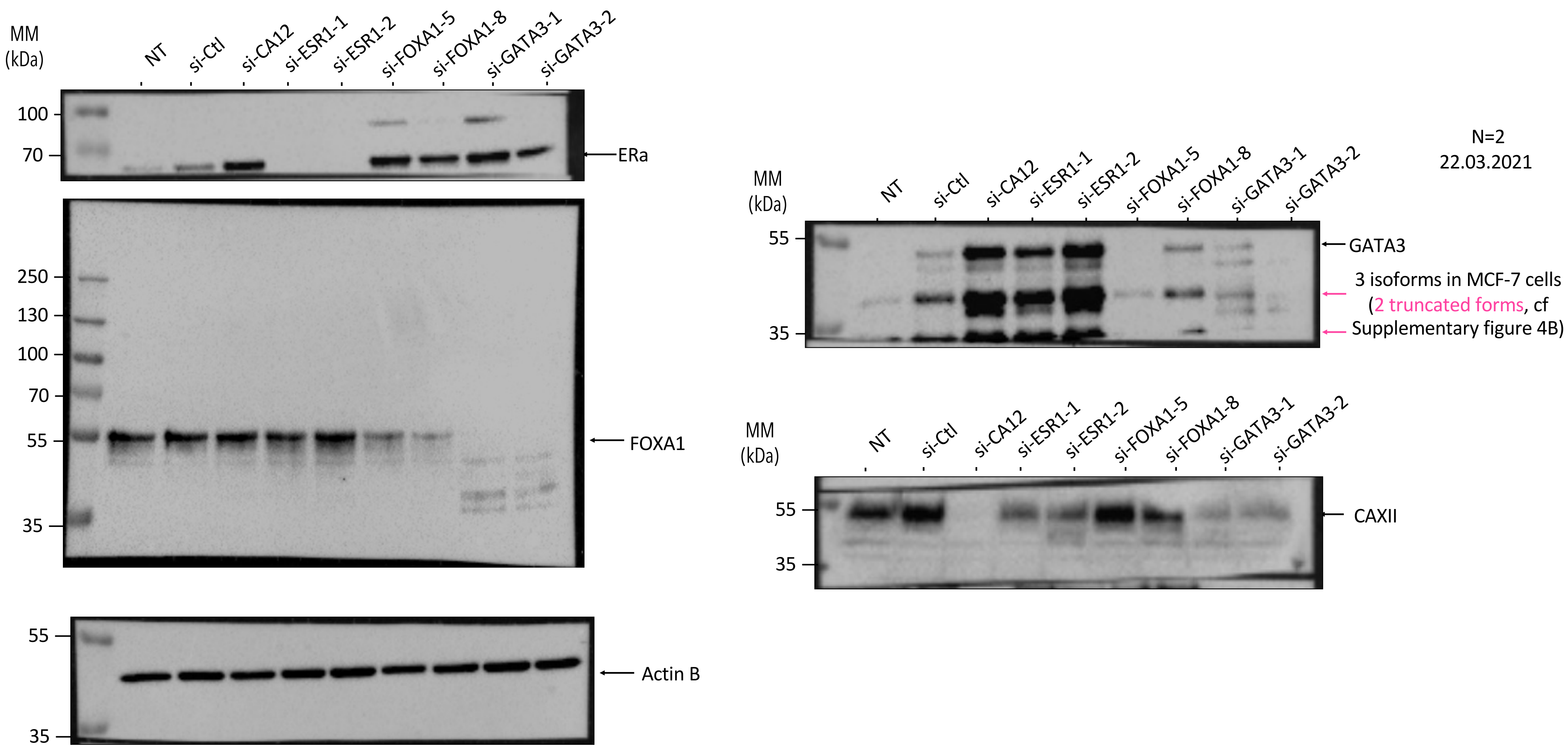

RPMI (T-47D, ZR-75) complemented with 10% of FBS

Gel SDS-PAGE : 8-10%

Loading quantity : 40µg

Standardisation : Lamin B1

Blocking with 5% milk in PBST (Tris Buffer Saline 1X + 0.1% tween 20) for 1h.

Primary Antibodies

rAb CA12 (44 kDa), Dr Sly, dilution 1/7000 T-47D/ZR-75

mAb GATA3 (48kDa), 558686 BD Pharmingen, dilution 1/1000

Actin B (42 kDa), dilution 1/10000.

Lamin B1 (66kDa), dilution 1/3000.

Secondary Antibody dilution, anti-rabbit/mouse (1:10000)

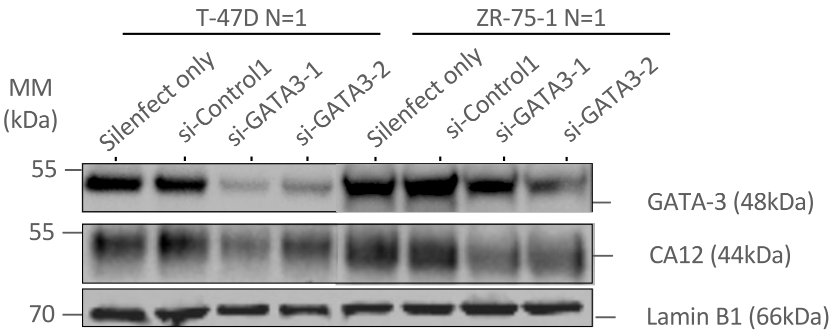

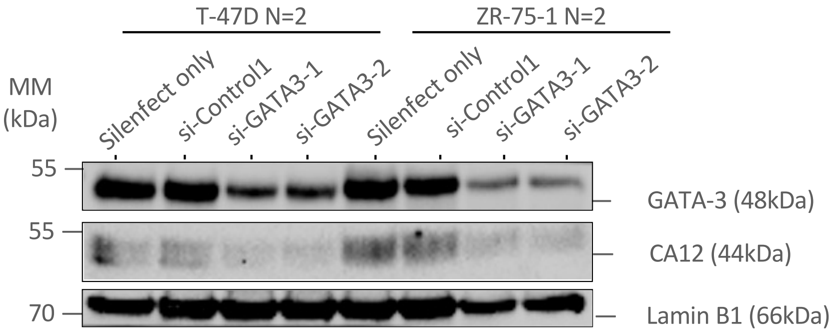

Figure 4 F-G

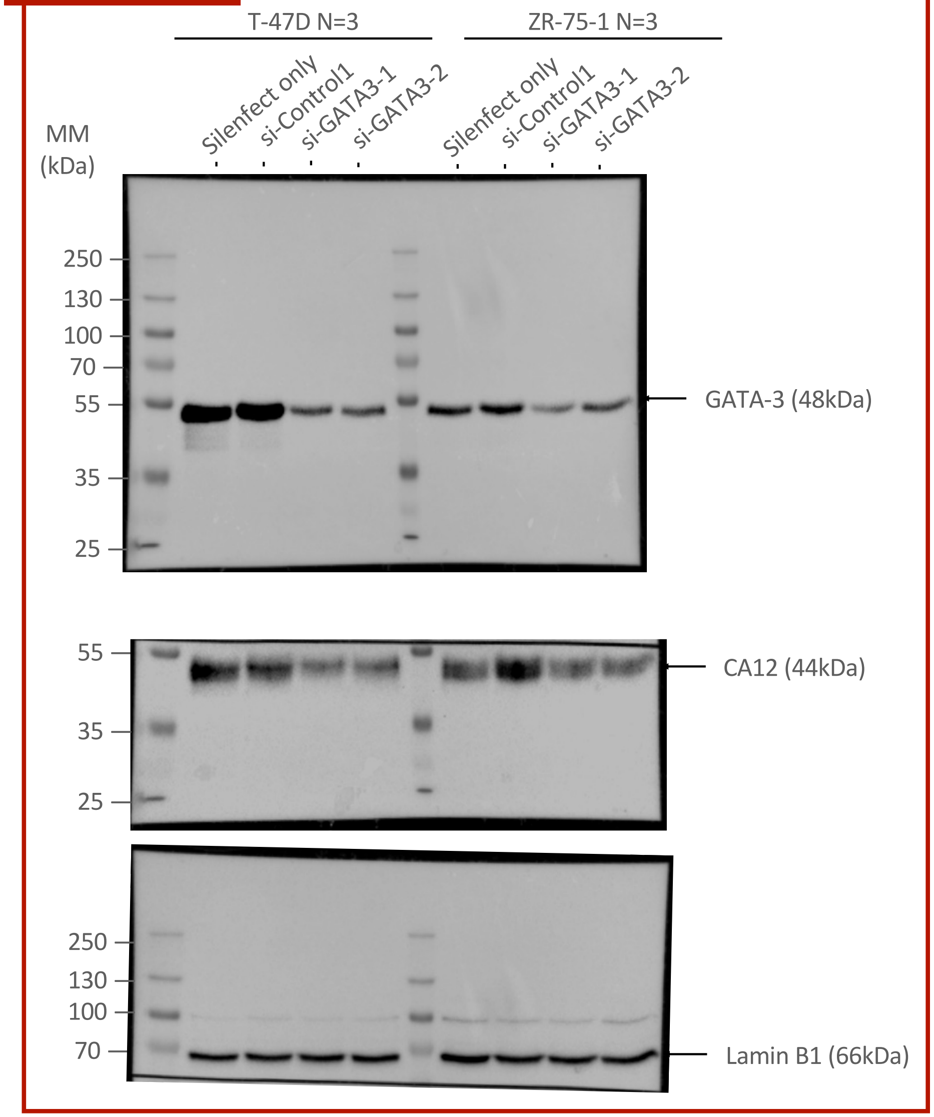

		Ratio si-GATA3/si-Control		
		N1	N2	N3
	si-Control	1	1	1
	si-GATA3-1	0.5189918	0.4443561	0.4381894
T-47D	si-GATA3-2	0.8887144	0.4074548	0.4713568
	si-Control	1	1	1
	si-GATA3-1	0.5718026	0.3456733	0.5053662
ZR-75-1	si-GATA3-2	0.6993163	0.2814324	0.4057448

Figure 4 F-G

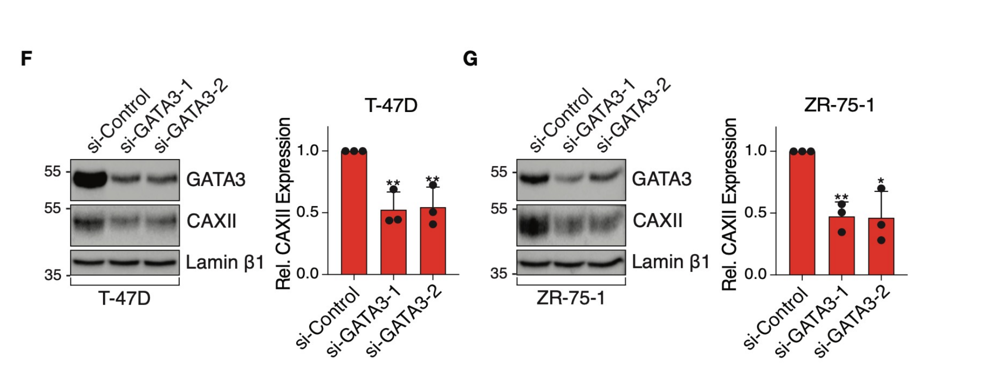

DMEM (MCF-7, SKBR-3, MDA-231, MDA-453), RPMI (T-47D, ZR-75 and HCC-70) and DMEM/F12 (MCF10A) complemented with 10% of FBS

Gel SDS-PAGE : 8-10%

Loading quantity : 40µg

Standardisation : Actin B

Blocking with 5% milk in PBST (Tris Buffer Saline 1X + 0.1% tween 20) for 1h.

Primary Antibodies

rAb CA12 (44 kDa), Dr Sly, dilution 1/7000
rAb ERa (66 kDa), 60-C anticorps, dilution 1/2000
rAb FOXA1 (55kDa), Ab170933, dilution 1/1000
mAb GATA3 (48kDa), 558686 BD Pharmingen, dilution 1/1000
mAb Actin B (42 kDa), dilution 1/10000.

Secondary Antibody dilution, anti-rabbit/mouse (1:10000)

Figure S4B

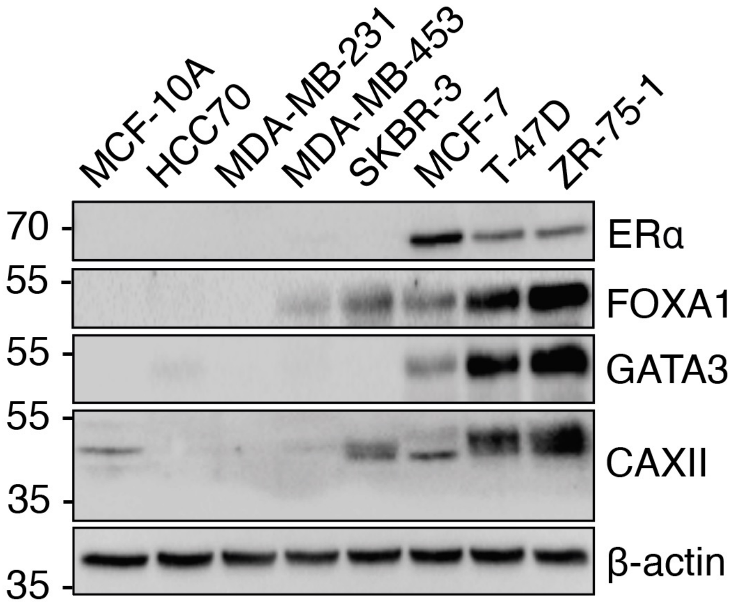

DMEM (MCF-7, SKBR-3, MDA-231, MDA-453), RPMI (T-47D, ZR-75 and HCC-70) and DMEM/F12 (MCF10A) complemented with 10% of FBS

Gel SDS-PAGE : 8-10%

Loading quantity : 40µg
Standardisation : Actin B

Blocking with 5% milk in PBST (Tris Buffer Saline 1X + 0.1% tween 20) for 1h.

Primary Antibodies
rAb CA12 (44 kDa), Dr Sly, dilution 1/7000
rAb ERa (66 kDa), 60-C anticorps, dilution 1/2000
rAb FOXA1 (55kDa), Ab170933, dilution 1/1000
mAb GATA3 (48kDa), 558686 BD Pharmingen, dilution 1/1000
mAb Actin B (42 kDa), dilution 1/10000.

Secondary Antibody dilution, anti-rabbit/mouse (1:10000)

Figure S4B

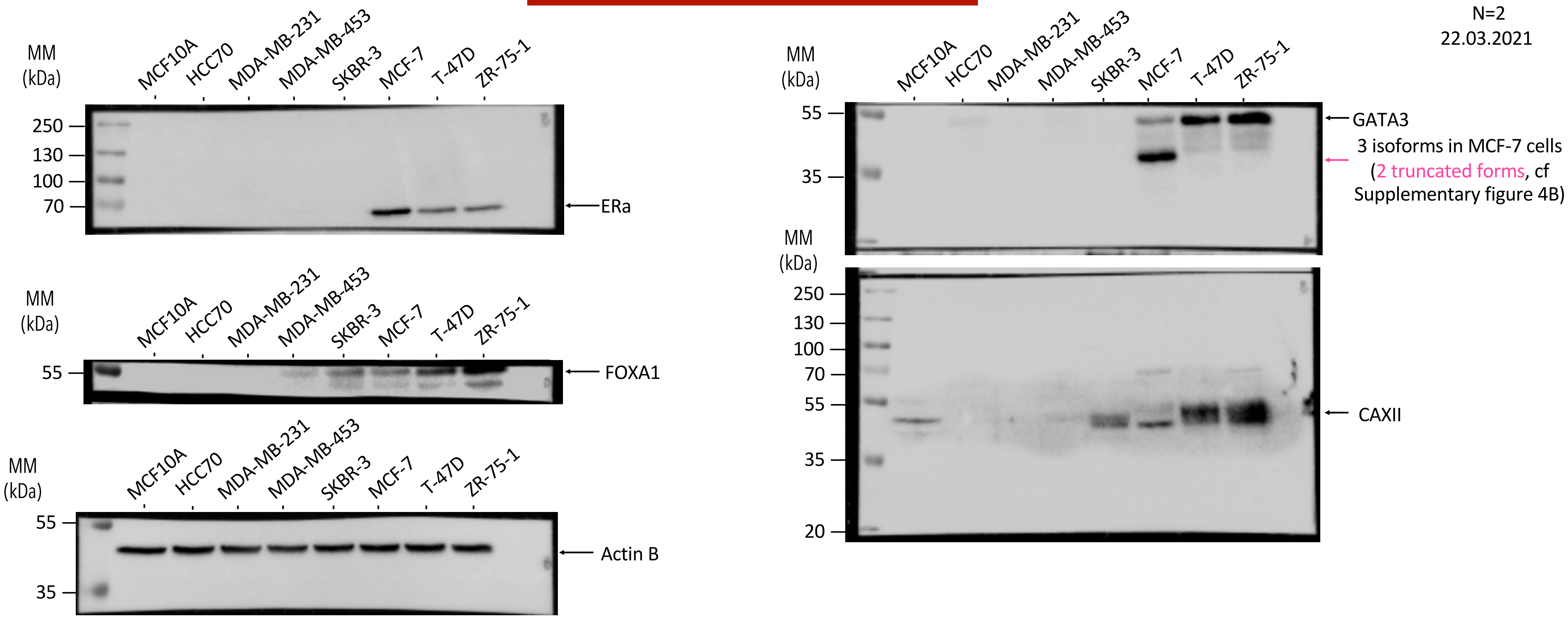

Supplement: Supplementary file 1 [file cancers-14-05453-s001.zip › cancers-1964955-File S1-original images.pdf]
