# Supplementary material for: CAXII Is a Surrogate Marker for Luminal Breast Tumors Regulated by ER and GATA3
Source: Cancers (Basel). 2022 Nov 6;14(21):5453. doi: 10.3390/cancers14215453 (PMC9657334; doi:10.3390/cancers14215453)
Supplement: Supplementary file 1 [file cancers-14-05453-s001.zip › cancers-1964955-Supplementary Figures&Tables.pdf]

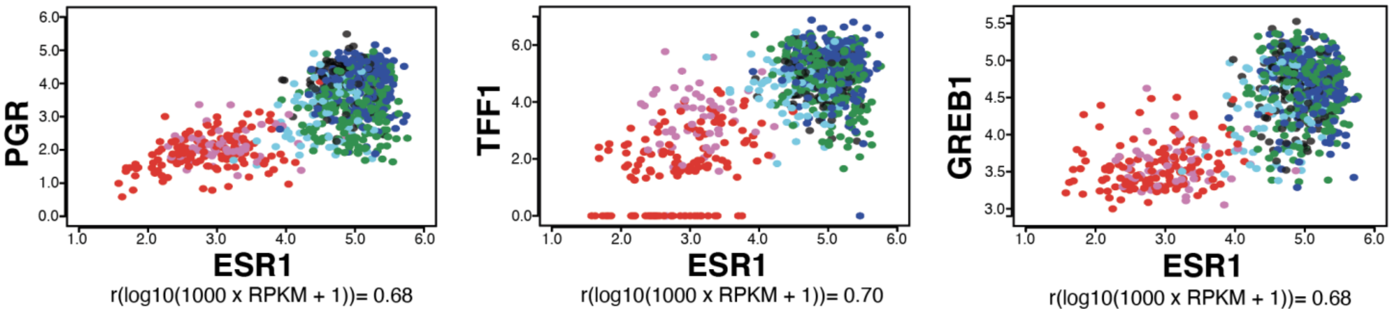

Figure S1. Correlations between mRNA levels of *ESR1* and of several estrogen target genes in breast cancer. Tumor subtypes according to the CIT classification are indicated by colors. Luminal A tumors appear in dark blue, luminal B in green, luminal C in light blue, molecular apocrine in magenta and basal-like in red. Normal-like or non-classified tumors are shown in black.

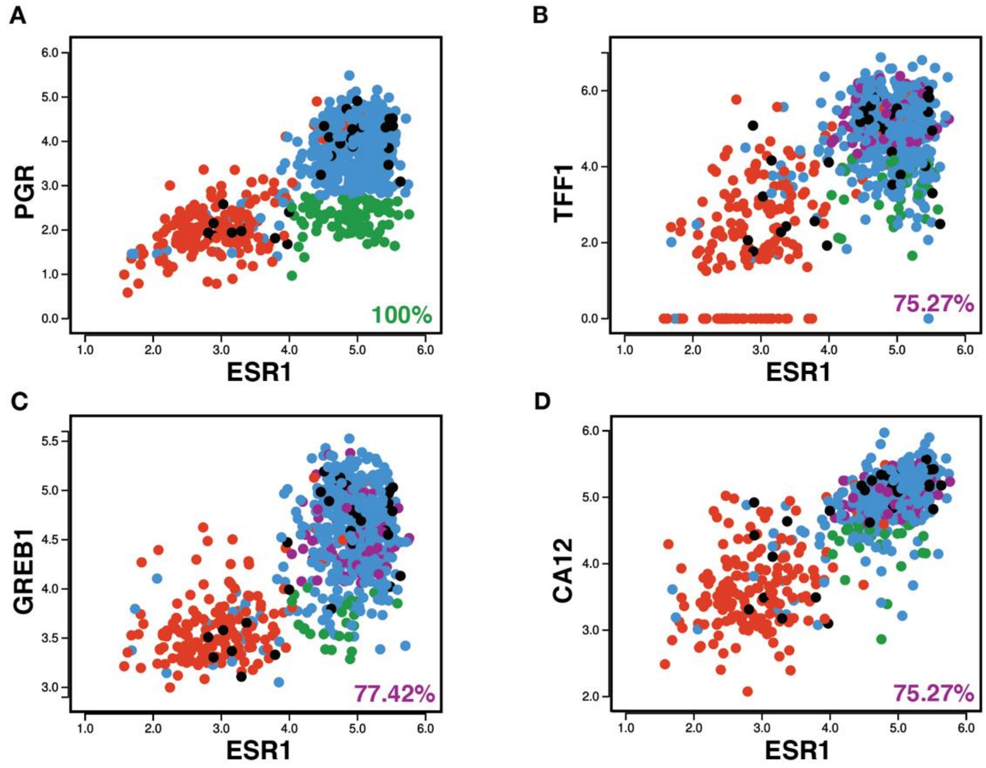

Figure S2. *ESR1*^{high}*PGR*^{low} status does not imply lack of ER signaling. Pair-wise scatterplots for the expression of *PGR* vs *ESR1* generated using MiSTIC [39] (http://mistic.iric.ca, accessed on 1 September 2022). Tumors highlighted in green (**A**) are *ESR1*^{high}*PGR*^{low} (PGR cut-off excluding 90% of *ESR1*^{low} tumors). The percentages of these tumors that are *TFF1*^{high} (**B**) *GREB1*^{high} (**C**) or *CA12*^{high} (**D**) using cut-offs for these genes that exclude 90% of *ESR1*^{low} tumors are indicated and the corresponding tumors are highlighted in purple.

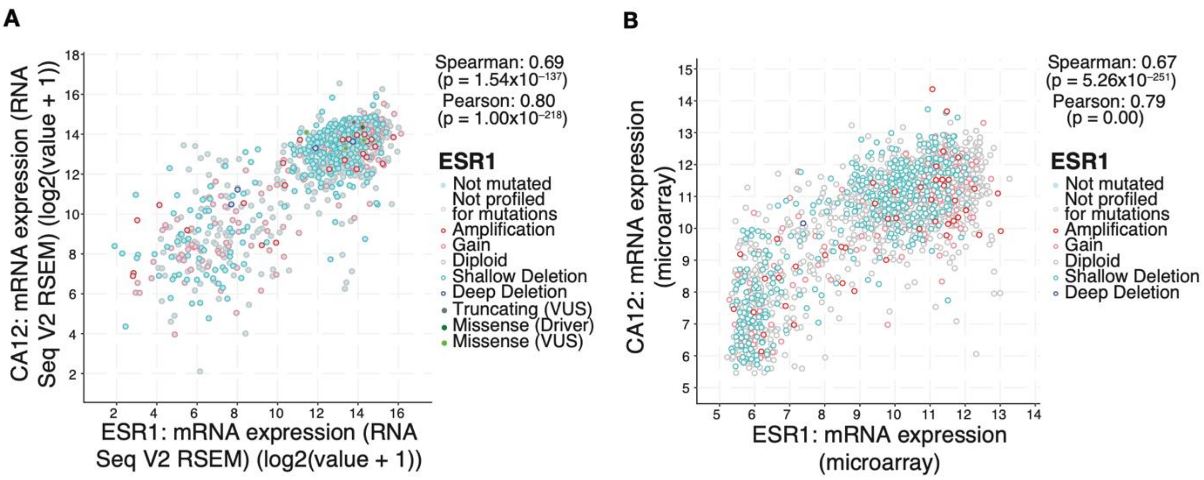

Figure S3. ***CA12* is mainly expressed in luminal tumors.** Correlation between *ESR1* and *CA12* mRNA expression levels in (**A**) the TCGA Firehose Legacy breast invasive carcinoma dataset (960 tumors) and (**B**) the METABRIC microarray dataset (1904 tumors). Data was generated from www.cbioportal.org, accessed on 1 September 2022. by querying the *CA12* and *ESR1* genes in the above-mentioned datasets.

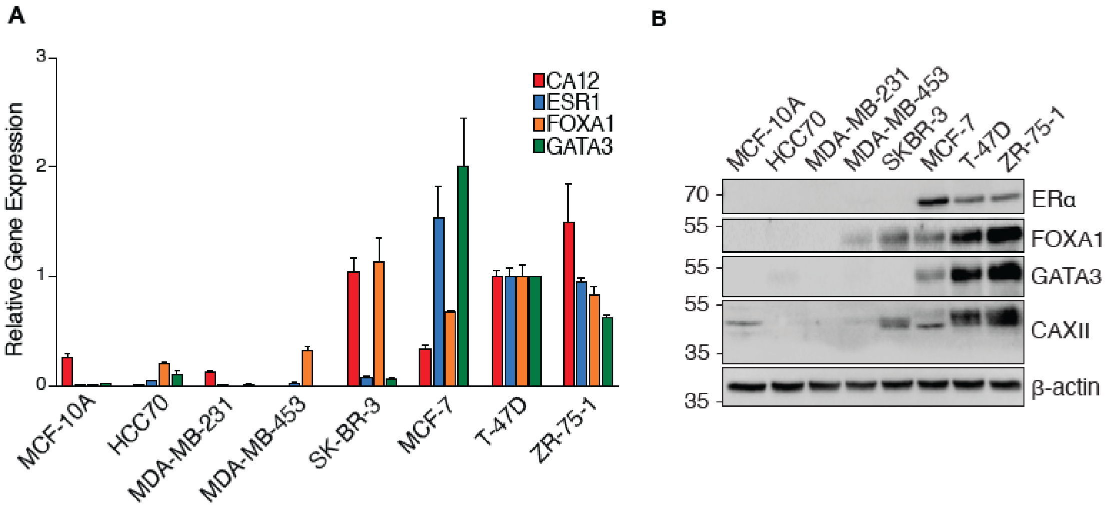

Figure S4. ***CA12* is highly expressed in luminal and in some mApo breast cancer cell lines.** MCF-10A, HCC-70, MDA-MB-231, MDA-MB-453, SKBR-3, MCF-7, T-47D and ZR-75-1 cell lines were cultured in hormone-replete medium and collected for mRNA and protein analysis. (**A**) *CA12*, *ESR1*, *FOXA1* and *GATA3* mRNA levels were measured by RT-qPCR (Representative N, N=3). *RPLP0* and *YWHAZ* were used as housekeeping genes for normalization. Expression levels are shown relative to those in T-47D cells. (**B**) Protein levels of ERα, FOXA1, GATA3, CAXII and β-actin were analyzed by western blotting using specific antibodies (N=3, a representative blot is shown). β-actin serves as a loading control.

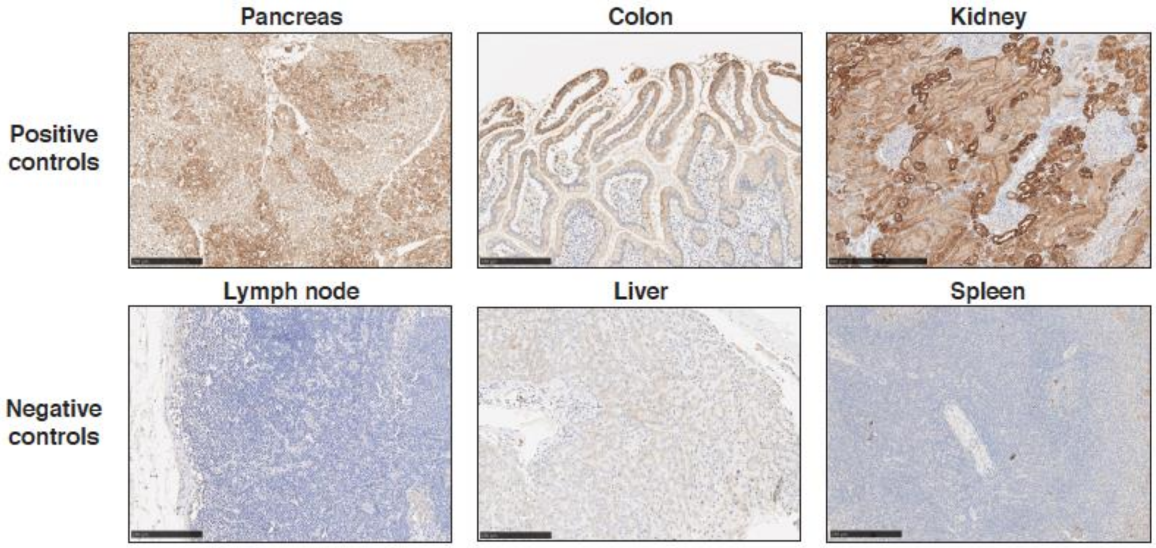

Figure S5. CAXII staining optimization. Pancreas, colon and kidney are positive controls for CAXII expression and lymph node, liver and spleen are negative controls.

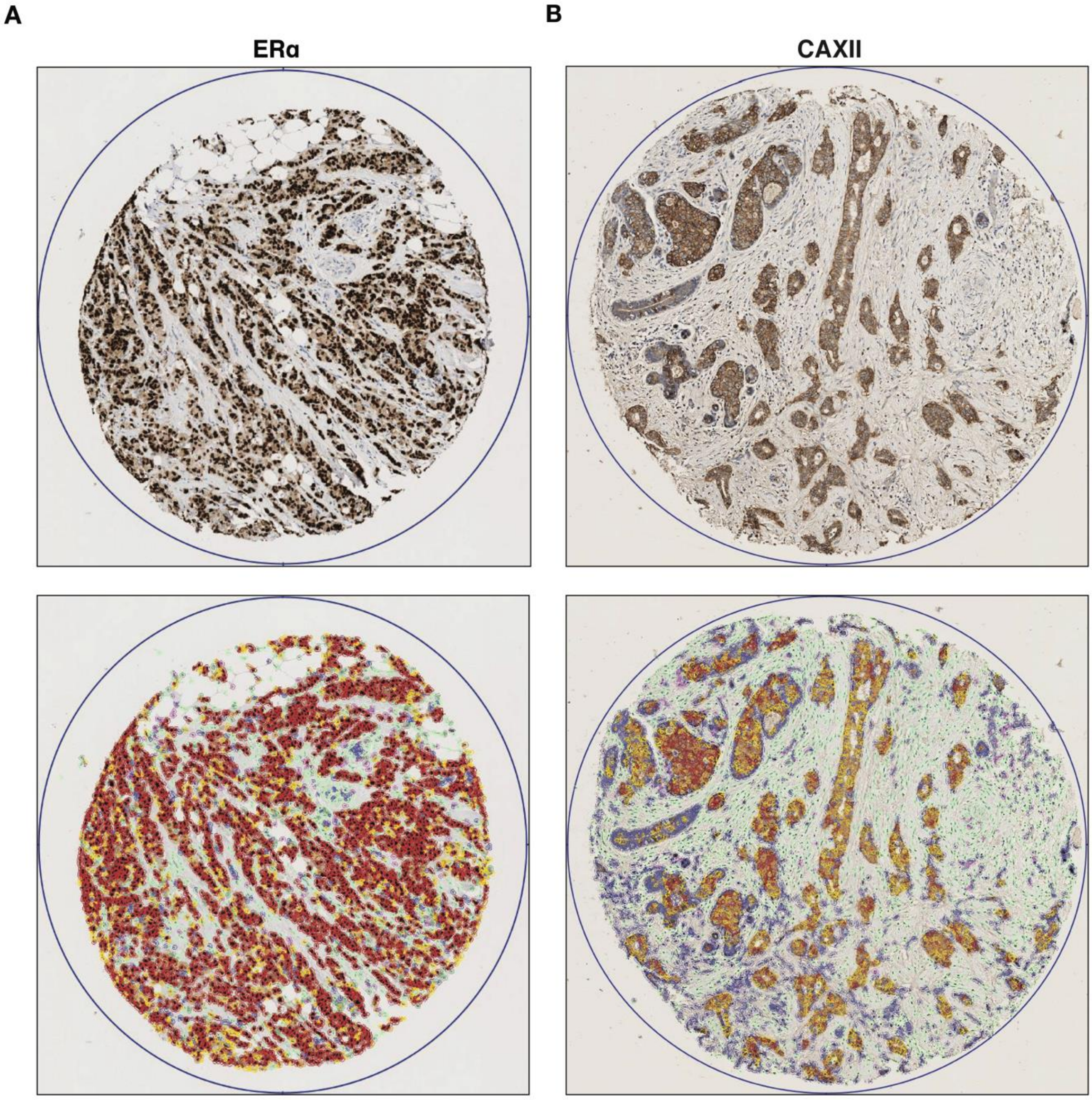

Figure S6. Tumor cell detection and scoring by QuPath in breast tumor sections. Cell type attribution and scoring of epithelial tumor cells on breast tumor sections stained for ERa (**A**) or CAXII (**B**) was assessed using QuPath. Purple cells correspond to immune cells, green cells to stromal cells, yellow, orange and red cells to increasing intensity of expression of each marker in epithelial cells.

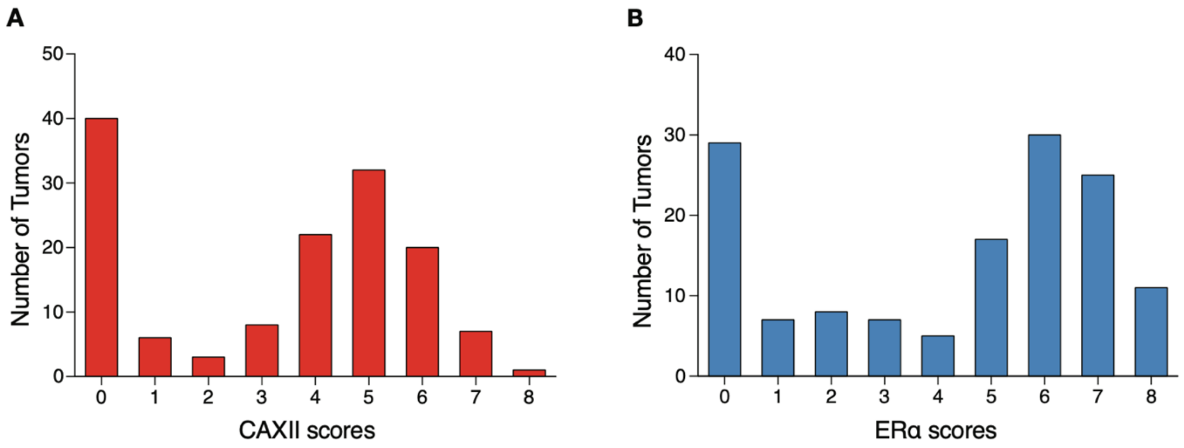

Figure S7. Bimodal distribution of CAXII and ERa QuPath scores. Histograms representing the number of tumors for each score. Scores (from 0 to 8) were generated with QuPath by analyzing the percentages of expressing cells and staining intensity for CAXII (**A**) and ERa (**B**).

Table S1. RT-qPCR primer sequences.

Gene	UPL probe number	Primer sequences
RPLP0 (Reference)	74	5'-TCCCACTTGCTGAAAAGGTC-3'
		5'-AGCAGGAGCAGCTGTGGT-3'
YWHAZ (Reference)	2	5'-GCAATTACTGAGAGACAACTTGACA-3'
		5'-TGGAAGGCCGGTTAATTTT-3'
CA12	11	5'-GCTCCTGCTGGTGATCTTAAA-3'
		5'-CCAAAATAAGTCCACTTGGAACC-3'
ESR1	25	5'-AAGAAACCGGATGGCAGTC-3'
		5'-CAGTGTGTGGTGGACTACGG-3'
FOXA1	47	5'-ATCATTGCCATCGTGTGCTT-3'
		5'-CACCATGTCCAACTGTGGAA-3'
GATA3	36	5'-ACTACGGAAACTCGGTCAGG-3'
		5'-GGTAGGGATCCATGAAGCAG-3'

Table S2. Immunoblotting and immunoprecipitation conditions.

	Reference	Provider	Species	Dilution for Western blotting	Quantity for ChIP-qPCR (μg)
CAXII	Ref 47	Drs Sly and Waheed	rabbit	1/3000	3
ER*α*	04-820 (clone 60C)	Millipore Sigma	rabbit	1/3000	3
FOXA1	HPA050505	Sigma-Aldrich	rabbit	1/1000	3
GATA3	558686	BD Pharmingen	mouse	1/3000	-
	ab199428	Abcam	rabbit	-	3
Lamin B1	ab16048	Abcam	rabbit	1/3000	-
b-actin	A5441	Sigma-Aldrich	rabbit	1/10000	-
IgG	3900S	Cell Signaling Technology	rabbit	-	3

Table S3. SiRNA oligonucleotide sequences.

Gene	Sequence
Control	UGGUUUACAUGUCGACUAA
CA12 (SMARTpool)	GAGGACCGCUGAAUAUUAC CCAUAGACCUGCACAGUGA CCAGAGAAAUGAUCAACAA GCUACAAUCUGUCUGCCAA
ESR1#1	GAAUGUGCCUGGCUAGAGAUU
ESR1#2	CAUGAGAGCUGCCAACCUUUU
FOXA1#1	GCACUGCAAUACUCGCCUUUU
FOXA1#2	CCUAAACACUUCCUAGCUCUU
GATA3#1	AAGCCUAAACGCGAUGGAUAUUU
GATA3#2	AACAUCGACGGUCAAGGCAACUU

Table S4. ChIP-qPCR primer sequences.

Gene	UPL probe #	Sequence
Desert region 1	82	5'-CCACCAGTGCATCCTCGAAT-3'
		5'-TTTCCCTTAGATGGACGGCG-3'
Desert region 2	22	5'-GATTCCAGAGTGTTCTGAGGCT-3'
		5'-GGTTAGTTGCCCCCTCGAAA-3'
CA12 enhancer 1	65	5′-TCTTCTCCACGCCCTGTAAA-3′
		5′-GACAGCTGCCAGGTACGG-3′
CA12 enhancer 2	60	5′-ACATCATGCCAGCTAGTTGACT-3′
		5′-TCTCTTGGAGGTCACAGTGC-3′
CA12 enhancer 3	38	5′-ATATCAGAGGTCCTGGGCCC-3′
		5′-TCAGGGAAAGTTCTAGCACCC-3′

Table S5. Clinicopathological characteristics of the 118 breast tumors.

Clinicopathological characteristics	Numbers (%)
Histological subtypes (118/118)	
Ductal carcinoma (IDC and DCIS)	109 (~92.4)
Lobular carcinoma	9 (~7.6)
Histological grade (115/118)	
Grade I	21 (~18.3)
Grade II	45 (~39.1)
Grade III	50 (~43.5)
Tumor types (118/118)	
ER+	72 (~61.0)
ER+PR+	55 (~46.6)
ER+PR-	17 (~14.4)
HER2+	9 (~7.6)
ER-PR-	9 (~7.6)
ER-HER2-	37 (~31.4)
ER-PR+HER2- (ER-PR+)	3 (~2.5)
ER-PR-HER2- (TN)	34 (~28.8)

Table S6. Antibodies and IHC staining conditions.

Antibody	Provider	Dilution	Staining	Epitope retrieval	Positive control	Negative control	Tested concentrations
CAXII	Drs Sly and Waheed (ref 47)	1/750	poly@Rb 15/0/8	H1(10)	mammary gland - pancreas - kidney - colon	lung - spleen - liver – lymph nodes	1/500 - 1/750 - 1/1000 (colon)
ERα (sc-8002) (0.2 mg/ml)	Santa Cruz Biotechnologies	1/1000	poly@Ms 30/15/15	H1(20)	endometrium - Fallopian tube - uterus - mammary gland	heart - tonsil - colon	1/500 - 1/750 - 1/1000 (mammary gland)
FOXA1 (HPA050505) (0.3 mg/ml)	Sigma-Aldrich	1/1000	poly@Rb 30/0/15	H1(20)	stomach - prostate - bladder - mammary gland	cerebral cortex - placenta – spleen	1-500 - 1/1000 - 1/500 poly@Rb 60/0/30 (tonsil - mammary gland)
GATA3 (RR8686) (0.5 mg/ml)	Bd Pharmingen	1/500	poly@Ms 30/15/15	H1(10)	rectum - urinary bladder - mammary gland	ovary - liver - neuro cerebellar cortex	1/100 - 1/250 - 1/500 - 1/1000 (urinary bladder)

Table S7. Co-IHC staining conditions.

Antibody	Dilution	Tissue
CAXII - ERα	1/750	Luminal ductal carcinoma comprising DCIS and IDC
	1/1000	
CAXII - FOXA1	1/750	
	1/500	
CAXII - GATA3	1/750	
	1/1000	
